# Phosphorescent Tris-cyclometalated Pt(IV) Complexes with Mesoionic *N*-Heterocyclic Carbene and 2-Arylpyridine Ligands

**DOI:** 10.1021/acs.inorgchem.2c02039

**Published:** 2022-07-21

**Authors:** Ángela Vivancos, Delia Bautista, Pablo González-Herrero

**Affiliations:** † Departamento de Química Inorgánica, Facultad de Química, Universidad de Murcia, Campus de Espinardo, 19, 30100 Murcia, Spain; ‡ Área Científica y Técnica de Investigación, Universidad de Murcia, Campus de Espinardo, 21, 30100 Murcia, Spain

## Abstract

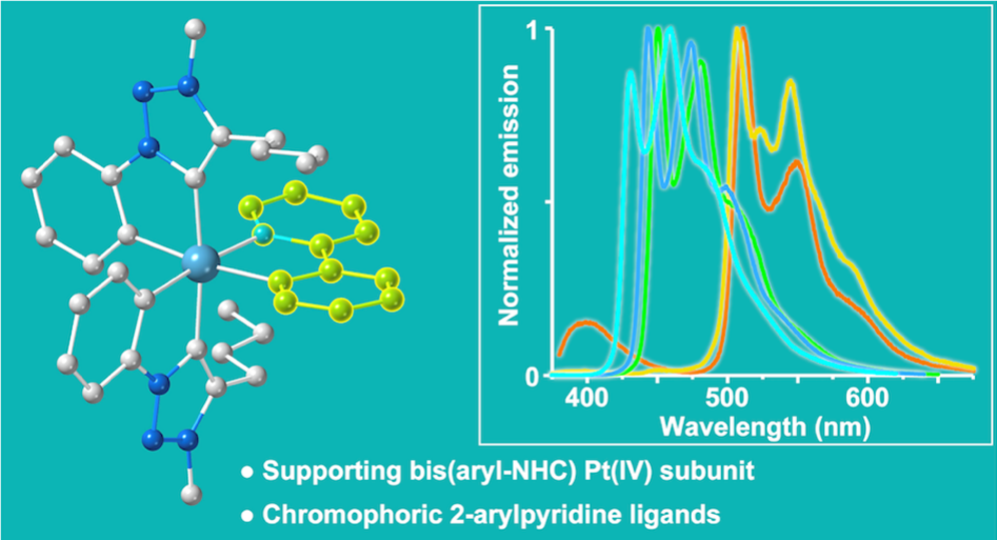

The synthesis, structure, photophysical properties, and electrochemistry of the first series of Pt(IV) tris-chelates bearing cyclometalated aryl-NHC ligands are reported. The complexes have the general formula [Pt(trz)_2_(C∧N)]^+^, combining two units of the cyclometalated, mesoionic aryl-NHC ligand 4-butyl-3-methyl-1-phenyl-1*H*-1,2,3-triazol-5-ylidene (trz) with a cyclometalated 2-arylpyridine [C∧N = 2-(2,4-difluorophenyl)pyridine (dfppy), 2-phenylpyridine (ppy), 2-(*p*-tolyl)pyridine (tpy), 2-(2-thienyl)pyridine (thpy), 2-(9,9-dimethylfluoren-2-yl)pyridine (flpy)], and presenting a *mer* arrangement or metalated aryls. They exhibit a significant photostability under UV irradiation and long-lived phosphorescence in the blue to yellow color range, arising from ^3^LC excited states involving the C∧N ligands, with quantum yields of up to 0.34 in fluid solution and 0.77 in the rigid matrix at 298 K. The time-dependent density functional theory (TD-DFT) calculations reveal that nonemissive, deactivating excited states of ligand-to-metal charge-transfer (LMCT) character are pushed to high energies as a consequence of the strong σ-donating ability of the carbenic moieties, making the Pt(trz)_2_ subunit an essential structural component that enables efficient emissions from the chromophoric C∧N ligands, with potential application for the development of different Pt(IV) emitters with tunable properties.

## Abstract 2

Tris-chelate Pt(IV) complexes that combine two cyclometalated mesoionic aryl-NHC ligands of the 1,2,3-triazolylidene subclass (C∧C*) with one cyclometalated 2-arylpyridine ligand (C∧N) are reported, which exhibit significant phosphorescent emissions that can be tuned by the variation of the C∧N ligand. The Pt(C∧C*)_2_ subunit is demonstrated as a useful platform for the development of efficient Pt(IV) emitters through the incorporation of different chromophoric ligands.

## Introduction

The use of N-heterocyclic carbene ligands (NHCs) for the design of strongly luminescent transition-metal complexes has become widespread, mostly associated with the development of phosphors for organic light-emitting devices (OLEDs).^1−4^ Mesoionic NHCs have received special attention within this field because their exceptionally strong σ-donating ability makes them particularly well suited to induce large ligand-field splittings, raising the energies of dissociative, metal-centered (MC) excited states and reducing the nonradiative deactivation that takes place through the thermal population of such states.^5−7^ This effect has even been applied with remarkable success to extend the excited-state lifetimes of strongly deactivated first-row transition-metal complexes.^8−11^

Bidentate cyclometalated aryl-substituted NHC ligands (aryl-NHCs, C∧C*) have been extraordinarily successful with the Ir(III)^12−21^ and Pt(II)^22−31^ ions as a replacement of cyclometalated 2-arylpyridines (C∧N), enabling better photostabilities, wider color tunability, and higher emission efficiencies. These enhancements are brought about by the larger ligand-field splitting induced by the NHC moiety with respect to the pyridine and, consequently, the reduced thermal accessibility of MC states from the triplet, mixed ligand-centered/metal-to-ligand charge-transfer (^3^LC/MLCT) emissive state of Ir(III) and Pt(II) complexes. The most relevant tris-chelates bearing C∧C* ligands are homoleptic Ir(III) complexes *mer*/*fac*-[Ir(C∧C*)_3_]^12,16,17,21^ and mixed-carbene variations,^19^ which can achieve blue phosphorescent emissions, thanks to the large π–π* gap of the ligands. Heteroleptic tris-chelates of the type [Ir(C∧C*)(C∧N)_2_] have also been reported, in which the arylcarbene acts as a supporting, nonchromophoric ligand, whereas the emission is mostly determined by the C∧N ligands,^13,18,32−34^ except for a few cases that incorporate C∧C* ligands featuring low π–π* gaps.^35−37^ However, very few heteroleptic tris-chelates bearing two C∧C* ligands are known, which include complexes [Ir(C*∧C∧C∧C*)(C∧N)] bearing a bis-aryl-NHC^38^ and [Ir(C∧C*)_2_(N∧N)] or [Ir(C∧C*)_2_(N∧N)]^+^, where N∧N is a pyridylpyrazolate, pyridyltriazolate, pyridylbenzimidazolate,^39−41^ or bipyridyl.^42^ Such systems are interesting because the Ir(C∧C*)_2_ subunit functions as a robust platform for the development of efficient emitters whose properties can be tuned by incorporating different chromophoric C∧N or N∧N ligands.

When compared with other d^6^ metal ions, the photophysical properties of Pt(IV) complexes have received much less attention. In previous studies, we have shown that cyclometalated Pt(IV) complexes with 2-arylpyridine ligands may exhibit very efficient and long-lived phosphorescence,^43−48^ which makes them potentially useful as luminescence-based sensors, photosensitizers, or photocatalysts. Their emissive excited states are essentially ^3^LC in character, with very little metal orbital participation in the form of an MLCT admixture. Because of the high oxidation state of the metal, unoccupied dσ* orbitals have relatively low energies and, in some derivatives, electronic promotions to these orbitals, i.e., ligand-to-metal charge-transfer (LMCT) excited states, may become thermally accessible from the emissive state. Such states have dissociative character because dσ* orbitals are strongly antibonding, providing a pathway for nonradiative deactivation or photochemical reactivity.^49,50^ Therefore, an indispensable requirement for Pt(IV) complexes to reach high emission efficiencies is the presence of suitable strong σ-donor ligands, which induce a large ligand-field splitting and raise the energy of LMCT states.

Recently, we reported Pt(IV) complexes of the types [PtCl_2_(C∧C*)(C∧N)] and [PtCl(C∧C*)(C∧N∧C)], where C∧C* is a cyclometalated, mesoionic aryl-NHC ligand of the 1,2,3-triazolylidene subclass, which constituted the first examples of Pt(IV) emitters bearing a carbene ligand.^51,52^ Derivatives of the type [PtCl_2_(C∧C*)(C∧N)] exhibited strong ^3^LC emissions involving the C∧N ligand, with significantly higher quantum efficiencies with respect to the homologous *C*_2_-symmetrical [PtCl_2_(C∧N)_2_] complexes as a consequence of the electronic effects of the carbene moiety. However, their synthesis presented problems associated with the difficult cyclometalation of the aryl-NHC ligand, resulting in relatively low yields. Herein, we present a straightforward methodology involving two consecutive cyclometalations of aryl-NHC ligands that has allowed the synthesis of complexes of the type [Pt(C∧C*)_2_(C∧N)]^+^. These species are the first Pt(IV) tris-chelates bearing cyclometalated aryl-NHC ligands and show intense phosphorescent emissions that can be modulated through the variation of the C∧N ligand, demonstrating the usefulness of the Pt(C∧C*)_2_ subunit as a platform for the design of efficient emitters.

## Results and Discussion

### Synthesis and Characterization

**Scheme 1:**
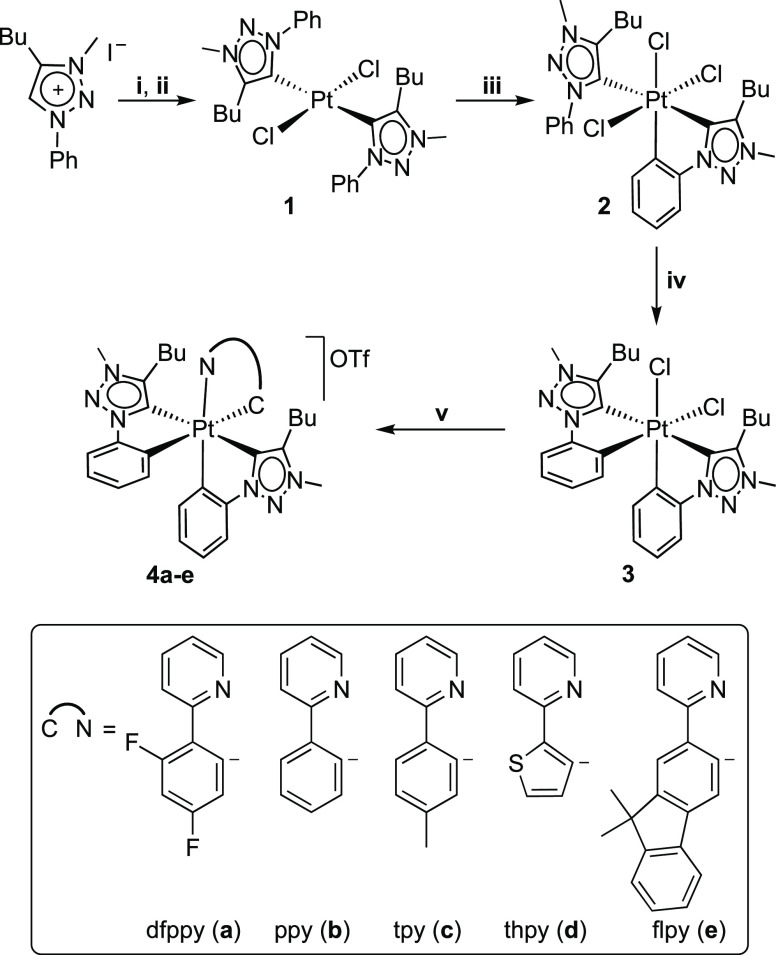
Synthetic Route to the Target Tris-cyclometalated Pt(IV) Complexes (i) Ag_2_O, 1,2-dichloroethane (DCE), 50 °C; (ii) (Pr_4_N)_2_[Pt_2_Cl_6_], DCE, 80 °C; (iii) PhICl_2_, CH_2_Cl_2_; (iv) Na_2_CO_3_, 1,2-dichlorobenzene (DCB), 120 °C; and (v) 2 AgOTf, N∧CH, DCB, 120 °C.

The synthetic route to the target tris-cyclometalated Pt(IV) complexes is shown in Scheme 1. The reaction of the dimeric platinum precursor (Pr_4_N)_2_[Pt_2_Cl_6_] with 4 molar equiv of the in situ-prepared silver carbene intermediate “AgI(trzH)” (trzH = 4-butyl-3-methyl-1-phenyl-1*H*-1,2,3-triazol-5-ylidene) in 1,2-dichloroethane at 80 °C led to the selective formation of *trans-*[PtCl_2_(trzH)_2_] (**1**), which could be isolated in 85% yield. The *trans* geometry of **1** was established from an X-ray diffraction analysis (see below). The reason for the exclusive formation of this isomer is probably that, upon coordination of the first trzH ligand, the Pt–Cl bond trans to the carbenic carbon becomes highly labile and is rapidly abstracted by the silver ion, resulting in the coordination of a second trzH ligand in this position. Consistent with this, the attempts to obtain a complex with only one trzH ligand by employing a 1:2 molar ratio (dimeric platinum precursor to carbene) were unsuccessful, resulting always in the formation of **1**. Similar results have been previously observed for the reactions of K_2_[PtCl_4_] with other silver carbenes.^53−57^

The ^1^H and ^13^C NMR spectra of complex **1** show two sets of resonances for the trzH ligand in very similar proportions (Figure S1), pointing to the presence of two different conformational isomers that interconvert very slowly at room temperature as a consequence of restricted rotation about the Pt–C bond. This possibility was confirmed by a variable-temperature NMR study in DMSO-d_6_, which showed that the different pairs of aromatic and aliphatic signals coalesce in the range 35–60 °C (Figure S2). Using the Eyring equation,^58^ a free energy of activation of Δ*G*^‡^ = 15.0 kcal/mol was calculated for this process at the coalescence temperature of the NCH_2_ protons (60 °C). Several examples of restricted rotation of NHC ligands about the metal–C bond have been previously reported.^53,59−61^

**Figure 1:**
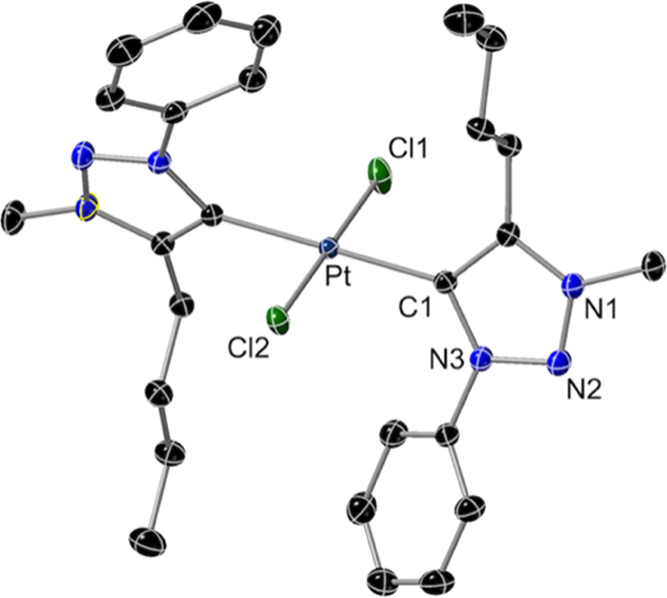
Structure of complex **1** (thermal ellipsoids at 50% probability). Hydrogen atoms are omitted. Selected bond distances (Å) and angles (°): Pt–C1, 2.027(2); Pt–Cl1, 2.3253(8); Pt–C12, 2.3132(7); C1–Pt–Cl1, 90.35(6); C1–Pt–Cl2, 89.65(6); and Cl1–Pt–C12, 180.0.

The crystal structure of **1** is shown in Figure 1. The crystal analyzed corresponded to the conformer with an antiparallel orientation of the phenyl and butyl substituents of the trzH ligands. The Pt–Cl bonds lie along a crystallographic 2-fold axis and therefore the coordination around the metal is strictly planar. The mean plane of the triazolylidene ring is rotated by 68.85° with respect to the coordination plane. The Pt–C1 bond distance of 2.027(2) Å is typical of Pt(II) complexes with mutually trans NHC ligands.^53−55,62−64^

**Figure 2:**
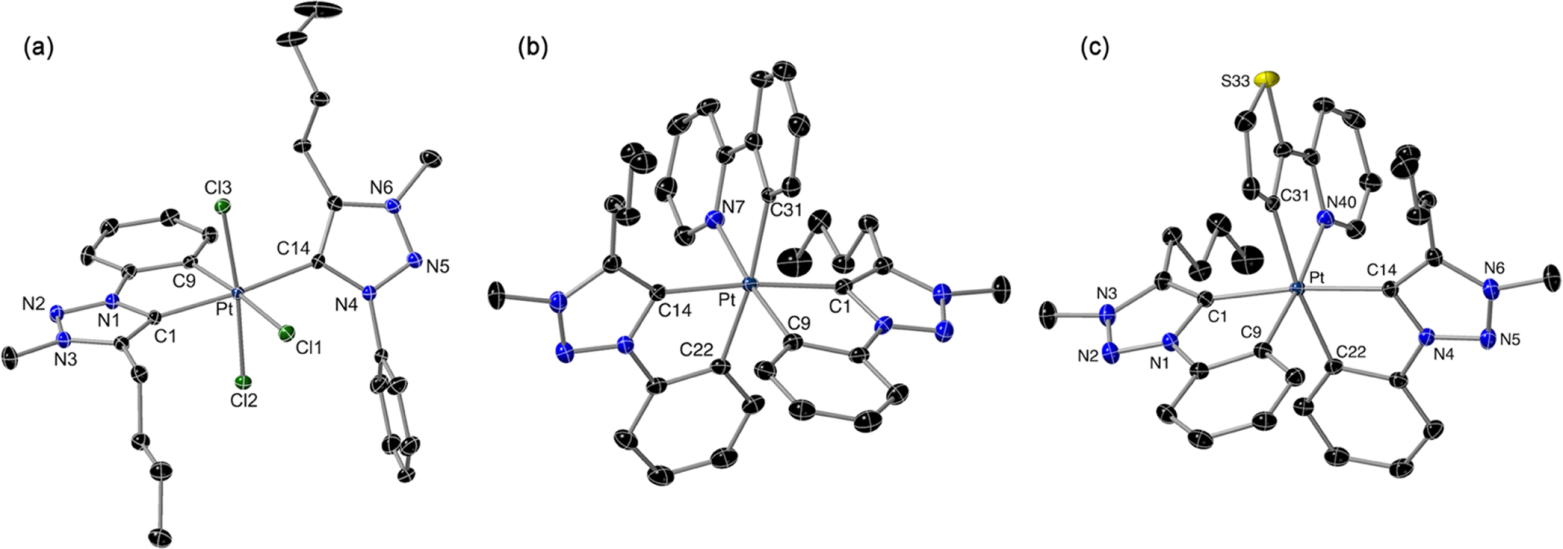
Structures of complexes **2** (a) and the cations of **4b** (b) and **4d** (c) (thermal ellipsoids at 50% probability). Hydrogen atoms are omitted. Selected bond distances (Å) and angles (°): **2**: Pt–C1, 2.046(2); Pt–C9, 2.032(2); Pt–C14, 2.089(2); Pt–Cl1, 2.4509(5); Pt–C12, 2.3190(5); Pt–Cl3, 2.3395(5); C1–Pt–C9, 80.95(8); and C1–Pt–C14, 173.90(8). **4b**: Pt–C1, 2.037(3); Pt–C9, 2.071(3); Pt–C14, 2.043(4), Pt–C22, 2.065(4); Pt–N7, 2.094(3); Pt–C31, 2.087(3); C1–Pt–C9, 80.22(14); C14–Pt–C22, 79.79(14); and N7–Pt–C31, 79.69(13). **4d**: Pt–C1, 2.034(2); Pt–C9, 2.0414(19); Pt–C14, 2.0393(19), Pt–C22, 2.0837(19); Pt–N40, 2.1164(17); Pt–C31, 2.0790(19); C1–Pt–C9, 80.06(8); C14–Pt–C22, 80.05(8); and N–Pt–C31, 79.46(7).

Treatment of a CH_2_Cl_2_ solution of **1** with PhICl_2_ led to the oxidation to Pt(IV) and the electrophilic metalation of the pendant aryl group of one of the trzH ligands, resulting in the formation of the monocyclometalated species [PtCl_3_(trz)(trzH)] (**2**), which was isolated in 96% yield. The ^1^H NMR spectrum of **2** shows an aromatic resonance flanked by ^195^Pt satellites at δ = 6.99 ppm (*J*_PtH_ = 37 Hz), arising from the proton ortho to the metalated carbon of a phenyl ring. The crystal structure (Figure 2) corroborated the presence of a cyclometalated trz and a coordinated trzH and revealed that the carbenic moieties remain mutually trans, resulting in a *mer* disposition of metalated carbons or chloride ligands.

The metalation of the pendant phenyl group of the remaining trzH ligand in **2** was achieved by heating at 120 °C in 1,2-dichlorobenzene in the presence of a base (Na_2_CO_3_), which resulted in the formation of the bis-cyclometalated complex [PtCl_2_(trz)_2_] (**3**, 78% yield). Its ^1^H NMR spectrum shows a single set of resonances arising from equivalent cyclometalated trz ligands. In addition, the proton ortho to the metalated carbon of the phenyl ring is strongly shielded (δ = 6.49 ppm, *J*_PtH_ = 52 Hz), indicating that it is affected by the diamagnetic current of an orthogonal aromatic ring. These data demonstrate a *C*_2_-symmetrical configuration with mutually cis chloride ligands.

The introduction of different cyclometalated 2-arylpyridines (C∧N) as the third chelating ligand was accomplished by reacting **3** with 2.4 equiv of AgOTf (OTf^–^ = trifluoromethanesulfonate) and an excess of the 2-arylpyridine in 1,2-dichlorobenzene at 120 °C, which afforded complexes [Pt(trz)_2_(C∧N)]OTf in 72–80% yields [C∧N = dfppy (**4a**), ppy (**4b**), tpy (**4c**), thpy (**4d**), flpy (**4e**)]. The ^1^H NMR spectra of these complexes show three distinctive aromatic resonances flanked by ^195^Pt satellites arising from the protons ortho to the metalated aryls, which are significantly shielded because they are directed toward orthogonal aromatic rings (δ = 7.04–6.29 ppm).

The crystal structures of complexes **4b** and **4d** were solved by X-ray diffraction analyses (Figure 2) and are completely analogous. The carbene moieties are mutually trans, and the coordinated nitrogen and metalated carbon of the C∧N ligand are trans to each of the metalated aryls of the trz ligands. Therefore, they retain the disposition of trz ligands found in their precursor, resulting in a *mer* configuration of metalated aryl rings. In the case of **4d**, the positions of the thiophene and pyridine rings were found disordered, with one of the orientations presenting a much higher occupancy factor than the other (*ca.* 81:19). Hence, the Pt–C9 or Pt–C22 bonds in **4d** can be considered as predominantly trans to the pyridyl or thienyl rings, respectively (Figure 2), the latter being significantly longer because of the stronger trans influence of the metalated carbon. However, in **4b** the positions of the pyridyl and phenyl rings of the ppy ligand were not distinguished by the refinement model, resulting in very similar Pt–C9 and Pt–C22 bond distances.

### Photophysical Properties

**Figure 3:**
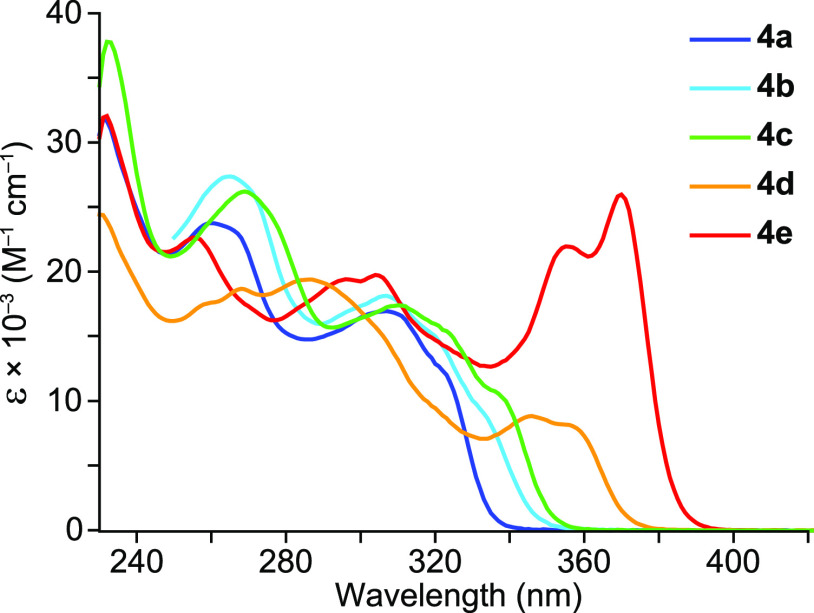
Electronic absorption spectra of complexes **4a–e** in a CH_2_Cl_2_ solution at 298 K.

The electronic absorption spectra of **4a–e** in CH_2_Cl_2_ solution (Table 1, Figure 3) show structured absorptions in the 250–400 nm range that can be ascribed to primarily ^1^LC transitions within the ligands. The shapes and energies of the observed bands are very similar to those of complexes [PtMe(Cl)(C∧N)_2_]^44^ and [PtCl_2_(trz)(C∧N)]^52^ with the respective 2-arylpyridine ligands, implying that the spectra are dominated by C∧N-centered absorptions, whereas those involving the trz ligands must be obscured. The lowest-energy absorption maximum shifts from 321 to 370 nm along the sequence **4a** → **4e**, as the expected energies of the lowest π–π* transition of the C∧N ligand decrease. As observed for [PtMe(Cl)(flpy)_2_],^44^ complex **4e** presents a significantly higher molar absorptivity (25900 M^–1^ cm^–1^) with respect to the rest of the derivatives (8100–12100 M^–1^ cm^–1^). Intense absorptions are advantageous for applications such as photocatalysis or bioimaging.

**Table 1:** Electronic Absorption Data for the Studied Complexes in CH_2_Cl_2_ Solution (*ca.* 5 × 10^–5^ M) at 298 K

complex	λ_max_ (nm) (ε × 10^–2^ (M^–1^ cm^–1^))
**4a**	260 (237), 307 (169), 321 (121)
**4b**	265 (274), 307 (181), 320 (149), 332 (91)
**4c**	268 (262), 310 (173), 323 (154), 337 (106)
**4d**	258 (174), 268 (187), 286 (194), 346 (88), 356 (81)
**4e**	256 (226), 296 (194), 304 (198), 355 (219), 370 (259)

**Figure 4:**
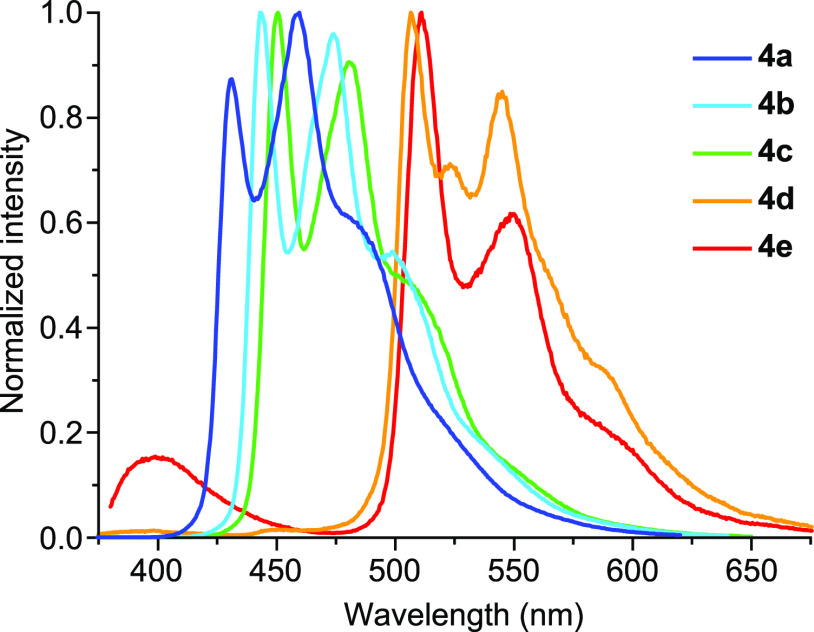
Emission spectra of complexes **4a–e** in CH_2_Cl_2_ at 298 K.

Before examining their luminescence, the photostability of **4a–e** was checked by irradiating their solutions in CD_3_CN in quartz NMR tubes with UV light (310 nm) for 6 h at room temperature. Only in the cases of **4a–c** were traces of decomposition products observed in the ^1^H NMR spectra (*ca.* 2% of the initial concentration; Figures S15–S19). This behavior is noteworthy because certain tris-chelate Pt(IV) complexes with a *mer* configuration of metalated aryl groups, *mer*-[Pt(C∧N)_3_]^+^ (C∧N = dfppy, ppy, tpy), isomerize to the *fac* complexes under iradiation with UV light as a consequence of the population of LMCT excited states.^43,45^ Instead, complexes **4a–e** produce significant luminescent emissions, which were characterized from deaerated CH_2_Cl_2_ solutions and poly(methyl methacrylate) (PMMA) matrices (2 wt %) at 298 K and frozen butyronitrile (PrCN) glasses at 77 K. The emission data at 298 K are summarized in Table 2, and the emission spectra in CH_2_Cl_2_ solution are shown in Figure 4. The data at 77 K and the complete series of excitation and emission spectra are included in the Supporting Information. Vibronically structured emissions are observed in all cases, characterized by large Stokes Shifts and lifetimes in the hundreds of microseconds range, which demonstrate a ^3^LC emissive state. Given that emission energies decrease in the same order as the lowest-energy absorption, the involved ligand is clearly the cyclometalated 2-arylpyridine, and therefore the trz ligands play a supporting role. In the case of the flpy derivative **4e**, a secondary emission band at a higher energy is assigned as fluorescence on the basis of its very short lifetime (<0.2 ns). This band represents a very small fraction of the emitted photons, with a quantum yield of Φ_F_ ≈ 0.005 in both CH_2_Cl_2_ and PMMA. We have previously reported dual fluorescent/phosphorescent emissions from Pt(IV) complexes bearing flpy^65^ or other C∧N ligands with extended π systems,^45,46^ which are due to a relatively less efficient intersystem crossing to the triplet manifold as a consequence of a lower metal orbital contribution to the involved excited states and the reduced spin–orbit coupling effects induced by the metal. Excitation spectra monitored at the phosphorescent emission band correlate with the corresponding absorption profiles in all cases. The excitation spectrum of **4e** monitored at the fluorescence band coincides with the one monitored at the phosphorescence band in the lower-energy region but shows some differences at higher energies that we tentatively attribute to relatively inefficient internal conversion between higher-lying ^1^LC(trz) states and the lowest ^1^LC(flpy) state (see Figure S21 for details). Compared with *fac*-[Pt(C∧N)_3_]^+^,^43^ [PtMe(Cl)(C∧N)_2_],^44^ and [PtCl_2_(trz)(C∧N)],^52^ the phosphorescent emissions of **4a–e** are somewhat blue-shifted, probably as a consequence of the stronger π-acceptor character of the trz ligands relative to 2-arylpyridines,^66^ leading to a lower energy of metal dπ orbitals and hence a lower MLCT contribution to the emissive excited state.

**Table 2:** Emission Data of Complexes **4a–e** at 298 K

complex	medium	λ_em_ (nm)^a^	Φ_P_^b^	τ (μs)^c^	*k*_r_ × 10^–3^ (s^–1^)^d^	*k*_nr_ × 10^–3^ (s^–1^)^e^
**4a**	CH_2_Cl_2_	431, 459, 486	0.34	118	2.9	5.6
	PMMA	431, 458, 486	0.68	348	1.9	0.9
**4b**	CH_2_Cl_2_	443, 474, 499	0.25	132	1.9	5.7
	PMMA	443, 473, 500	0.77	341	2.3	0.7
**4c**	CH_2_Cl_2_	450, 481, 507	0.24	169	1.4	4.5
	PMMA	450, 480, 508	0.77	433	1.8	0.5
**4d**	CH_2_Cl_2_	507, 523, 545, 588	0.15	148	1.0	5.7
	PMMA	507, 523, 545, 589	0.44	514	0.9	1.1
**4e**	CH_2_Cl_2_	400, 511, 550, 592	0.10	117	0.8	7.7
	PMMA	395, 509, 547, 592	0.39	750	0.5	0.8

a The most intense peak is italicized.

b Phosphorescence quantum yield.

c Lifetime.

d Radiative rate constant, *k*_r_ = Φ_P_/τ.

e Nonradiative rate constant, *k*_nr_ = (1 – Φ_P_)/τ.

Quantum yields vary in the range 0.10–0.34 in CH_2_Cl_2_ and 0.39–0.77 in PMMA matrix and reach the highest values for the derivatives bearing a ppy-based ligand (**4a–c**). The only previously reported luminescent Pt(IV) tris-chelates with a *mer* arrangement of metalated aryl rings contain at least one C∧N ligand of a relatively low energy for the π–π* transition, namely, *mer*-[Pt(flpy)_3_]^+^^65^ and the heteroleptic derivatives *mer*-[Pt(ppy)_2_(flpy)]^+^^65^ and *mer*-[Pt(C∧N)_2_(C′∧N′)]^+^ with C∧N = dfppy, ppy, C′∧N′ = thpy, 1-phenylisoquinoline (piq),^45^ and their quantum yields were in the range from 0.03 (for *mer*-[Pt(ppy)_2_(piq)]^+^) to 0.08 (for *mer*-[Pt(flpy)_3_]^+^).

The radiative and nonradiative rate constants (*k*_r_ and *k*_nr_, respectively) for the phosphorescent emissions were calculated assuming that the triplet emissive state is formed with unit efficiency. This assumption introduces a negligible error in the case of **4e** because the fluorescence emission has a very low quantum yield.^65^ The *k*_r_ values are similar to those of complexes *fac*-[Pt(C∧N)_3_]^+^ and [PtCl_2_(trz)(C∧N)] with the same C∧N ligands. The lower quantum yields of **4d** and **4e** are mainly attributable to their lower radiative rates, which are typically found for Pt(IV) complexes bearing thpy^45,46,52^ and flppy^65^ ligands and can be explained by a relatively poor metal-ligand orbital overlap, leading to decreased MLCT contributions to the emissive state. The *k*_nr_ values are drastically reduced in PMMA matrix in all cases, resulting in significantly higher quantum yields. This indicates that nonradiative deactivation in CH_2_Cl_2_ solution occurs mainly through molecular motion and collisions with solvent molecules.

### Electrochemistry

**Figure 5:**
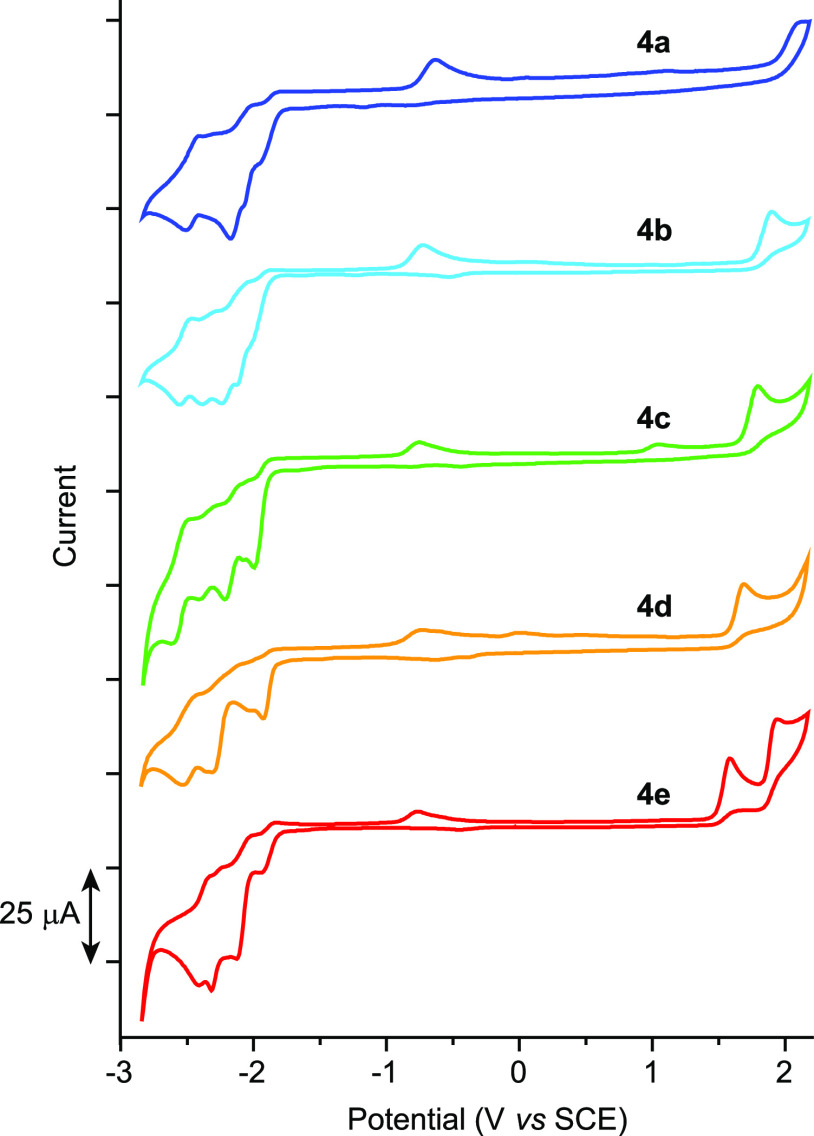
Cyclic voltammograms of complexes **4a**–**e** in MeCN at 100 mV s^–1^.

The cyclic voltammograms of complexes **4a**–**e** were registered in MeCN solution and are shown in Figure 5. The potentials of the observed redox processes and estimations of highest occupied/lowest unoccupied molecular orbital (HOMO/LUMO) energies are listed in Table 3. A single irreversible oxidation wave is observable within the accessible potential window for all complexes except **4e**, which produces two irreversible waves. The anodic peak potentials decrease in the sequence **4a** → **4e**, corresponding to increasing HOMO energies as the C∧N ligand becomes more electron donating. Therefore, the HOMO is essentially a π orbital of the C∧N ligand in all cases, which agrees with the density functional theory (DFT) calculations on **4c** (see below). The estimated HOMO energies are similar to those of other Pt(IV) complexes with the respective C∧N ligand in a similar coordination environment, *i.e.*, homoleptic *mer*-[Pt(C∧N)_3_]^+^ or heteroleptic *mer*-[Pt(C∧N)_2_(C′∧N′)]^+^ complexes,^45^ whereas the *fac* isomers^43,46,65^ and the bis-cyclometalated complexes [PtCl_2_(C∧N)(trz)]^52^ show lower values.

**Table 3:** Electrochemical Data^a^ and HOMO/LUMO Energy Estimations^b^ for Complexes **4a–e**

complex	*E*_p,a_^c^	*E*_p,c_^d^	*E*_1/2_^e^	*E*_HOMO_	*E*_LUMO_	Δ*E*_HOMO-LUMO_
**4a**	2.11	–1.92, −2.17	–2.04, −2.46	–6.63	–2.90	3.73
**4b**	1.89	–1.99, −2.23	–2.08, −2.35, −2.52	–6.46	–2.82	3.64
**4c**	1.79	–1.99	–2.16, −2.34, −2.55	–6.37	–2.81	3.56
**4d**	1.68	–1.92, −2.03, −2.29	–2.48	–6.28	–2.86	3.42
**4e**	1.58, 1.94		–1.88, −2.07, −2.27, −2.39	–6.18	–2.88	3.30

a In V vs SCE, registered in a 0.1 M solution of (Bu_4_N)PF_6_ in dry MeCN at 100 mV s^–1^.

b In eV.

c Irreversible anodic peak potentials.

d Irreversible cathodic peak potentials.

e For the reversible waves.

The first reduction wave is observed at very similar potentials for all complexes and is irreversible, except for **4e**, which shows a quasi-reversible wave (Figure S24). Additional reversible processes are observed at more negative potentials, corresponding to the reduction and subsequent reoxidation of species arising from the first reduction. The very similar LUMO energies indicate that this orbital is the same for all complexes and is not affected by the C∧N ligand. On the basis of DFT calculations on complex **4c**, the LUMO is composed of the combined lowest π* orbitals of the trz ligands.

### Computational Study

**Figure 6:**
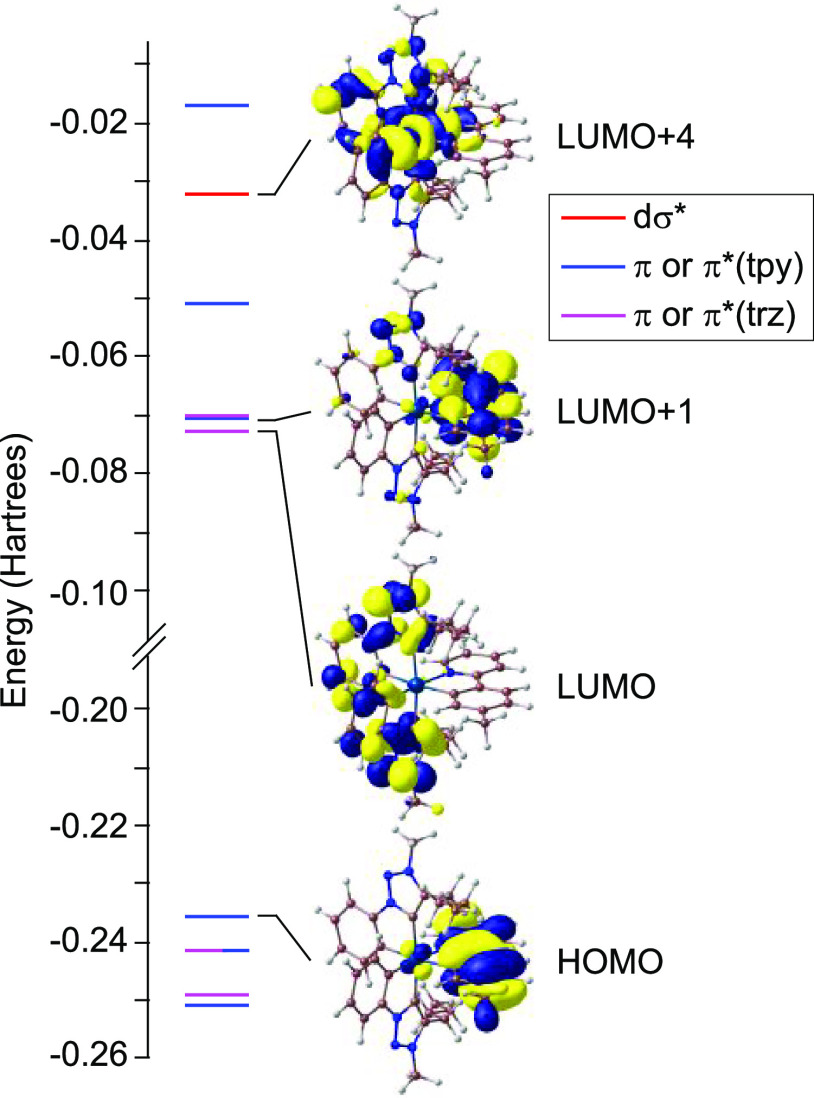
Molecular orbital energy diagram from DFT calculations and selected isosurfaces for complex **4c**.

For a more precise understanding of the properties of complexes **4**, DFT and time-dependent DFT (TD-DFT) calculations have been carried out for the tpy derivative **4c**. Complete details are presented in the Supporting Information, including fragment contributions to the frontier orbitals (Table S2). Figure 6 shows an orbital energy diagram, including selected isosurfaces. The HOMO is mainly composed of the highest π orbital of the tpy ligand with some dπ orbital contribution from the metal (*ca.* 3%), whereas the LUMO is made of the lowest π* orbital of the trz ligands and is similarly distributed over them. The LUMO+1 is the lowest π* orbital of the tpy ligand. The lowest molecular orbital with dσ* character is LUMO+4.

The TD-DFT results predict essentially LC transitions within the tpy ligand as the most intense, lowest-energy singlet excitations (Table S3), in agreement with the above interpretation of the experimental absorption spectrum. Analogous transitions involving the trz ligand are predicted at higher energies and have lower oscillator strengths. Ligand-to-ligand charge-transfer (LLCT) transitions between the tpy and trz ligands are predicted to occur at low energies, but their oscillator strengths are extremely low and therefore they cannot be identified in the experimental spectrum. The first three triplet excitations correspond to LC transitions within each of the cyclometalated ligands (Table S4), the lowest one involving the tpy, which is consistent with the assignment of the emissive state. The lowest triplet LMCT excitation involving an electronic promotion to LUMO+4 is 1.26 eV above the emissive state (T_15_, Table S4; *cf.* 0.64 or 0.78 eV for the lowest ^3^LMCT excitation in [PtCl_2_(tpy)_2_] or [PtCl_2_(trz)(tpy)], respectively),^52^ and is not expected to significantly contribute to nonradiative excited-state decay through thermal population.

**Figure 7:**
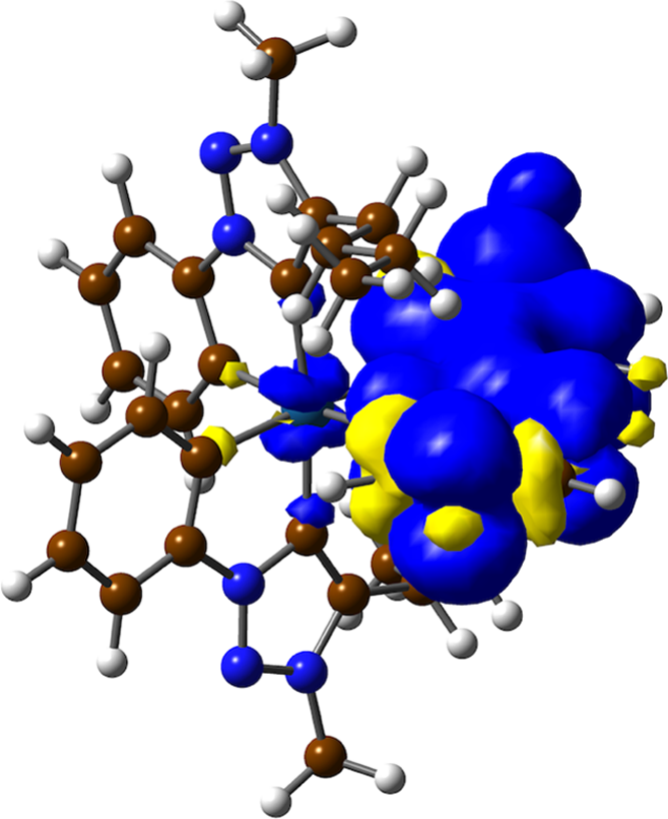
Spin density distribution (0.0008 e bohr^–3^) of the optimized lowest triplet excited state of **4c**.

A geometry optimization of the lowest triplet excited state of **4c** was carried out for additional insight. The calculated spin density distribution (Figure 7) essentially corresponds to a π–π* transition within the tpy ligand, which agrees with the above assignment of the emissive state as a primarily ^3^LC(tpy) excited state. The calculated natural spin density of 0.010 on the metal atom is comparable to those found for heteroleptic complexes of the type *mer-*[Pt(C∧N)_2_(C′∧N′)]^+^ (range 0.004–0.009)^45,65^ and can be interpreted as a very small MLCT contribution to the excited state. The adiabatic T_1_–S_0_ energy difference is 2.68 eV (463 nm), in good agreement with the observed emission energy.

## Conclusions

A method to obtain the *C*_2_-symmetrical, bis-cyclometalated species [PtCl_2_(trz)_2_] (**3**) has been developed, involving an oxidation of the Pt(II) bis-carbene complex, *trans*-[PtCl_2_(trzH)_2_] (**1**), and the successive electrophilic metalations of the pendant phenyl groups of the trzH ligands. Complex **3** is an excellent precursor for the preparation of cationic tris-cyclometalated complexes [Pt(trz)_2_(C∧N)]OTf (**4**) via chloride abstraction with AgOTf in the presence of 2-arylpyridine ligands. These are the first reported Pt(IV) tris-chelates bearing cyclometalated aryl-NHC ligands. Despite presenting a *mer* configuration of metalated aryls, they are significantly photostable under irradiation with UV light, in sharp contrast with most of the homologous *mer*-[Pt(C∧N)_3_]OTf complexes with the same C∧N ligands, which undergo photoisomerization reactions. In addition, they show significant phosphorescent emissions in different media, arising from ^3^LC states involving the C∧N ligands. The computational results show that the energies of deactivating ^3^LMCT states are high enough not to have an adverse effect on the emissions via thermal population from the emissive state, which is consistent with the observed photostabilities and can be explained by the strong σ-donation from the NHC moieties. Hence, the Pt(trz)_2_ subunit is demonstrated as a suitable platform upon which phosphorescent Pt(IV) tris-chelates with tunable emission energies can be built by incorporating different cyclometalated 2-arylpyridines as chromophoric ligands, opening the way to the development of new classes of emissive Pt(IV) complexes by employing other chromophoric bidentate ligands.

## Experimental Section

### General Considerations and Instrumentation

Preparations were carried out under atmospheric conditions, except for those that required silver reagents, which were conducted in the dark under an N_2_ atmosphere. Synthesis grade solvents were employed in all cases. The triazolium iodide salt,^67^ (Pr_4_N)_2_[Pt_2_Cl_6_],^68^ and PhICl_2_^69^ were synthesized according to reported procedures. All other reagents were obtained from commercial sources. Elemental analyses were carried out with a LECO CHNS-932 microanalyzer. Electrospray ionization high-resolution mass spectra (ESI-HRMS) were recorded on an Agilent 6220 Accurate-Mass time-of-flight (TOF) LC/MS. NMR spectra were registered on a 600 MHz Bruker Avance spectrometer at 298 K. The variable-temperature NMR spectra of **1** were registered on a 300 MHz Bruker Avance spectrometer. Chemical shifts (δ) were referenced to residual signals of nondeuterated solvent and are given in ppm downfield from tetramethylsilane. Abbreviations: s, singlet: d, doublet; t, triplet, q, quartet; p, pentet; sext, sextet; and m, multiplet.

#### Preparation of *trans*-[PtCl_2_(trzH)_2_] (**1**)

The triazolium salt (250 mg, 0.73 mmol) and Ag_2_O (110 mg, 0.47 mmol) were mixed in 1,2-dichloroethane (15 mL), and the resulting suspension was stirred at 50 °C for 14 h and then filtered through Celite. (NPr_4_)_2_[Pt_2_Cl_6_] (178 mg, 0.18 mmol) was added to the filtrate, and the mixture was stirred for 14 h at 80 °C and filtered through Celite. The filtrate was evaporated to dryness, and the residue was triturated with MeOH (3 × 4 mL) to give a white solid, which was recrystallized from CH_2_Cl_2_/Et_2_O and vacuum-dried to give **1**. Yield: 217 mg (85%). ^1^H NMR (600 MHz, CD_2_Cl_2_): δ = 8.47 (d, *J*_HH_ = 7.8 Hz, 2H, CH), 8.29 (d, *J*_HH_ = 7.8 Hz, 2H, CH), 7.58–7.51 (m, 4H, CH), 7.43 (t, *J*_HH_ = 7.7 Hz, 2H, CH), 4.05 (s, 3H, NCH_3_), 4.04 (s, 3H, NCH_3_), 3.22 (t, *J*_HH_ = 8.0 Hz, 2H, CH_2_), 3.04 (m, *J*_HH_ = 8.0 Hz, 2H, CH_2_), 2.01 (p, *J*_HH_ = 7.7 Hz, 2H, CH_2_), 1.79 (p, *J*_HH_ = 7.7 Hz, 2H, CH_2_), 1.57 (sext, *J*_HH_ = 7.3 Hz, 2H, CH_2_), 1.45 (sext, *J*_HH_ = 7.4 Hz, 2H, CH_2_), 1.04 (t, *J*_HH_ = 7.4 Hz, 3H, CH_3_), 0.97 (t, *J*_HH_ = 7.4 Hz, 2H, CH_3_). ^13^C APT NMR (150 MHz, CD_2_Cl_2_): δ = 157.3 (C), 157.0 (C), 147.2 (C), 147.0 (C), 140.7 (C), 140.5 (C), 129.6 (CH), 129.4 (CH), 129.3 (CH), 129.0 (CH), 125.5 (CH), 125.2 (CH), 36.6 (2 NCH_3_), 31.9 (CH_2_), 31.7 (CH_2_), 25.7 (CH_2_), 25.4 (CH_2_), 23.5 (CH_2_), 23.4 (CH_2_), 14.34 (CH_3_), 14.29 (CH_3_). Anal. Calcd for C_26_H_34_Cl_2_N_6_Pt: C, 44.83; H, 4.92; N, 12.06. Found: C, 44.71, H, 4.72, N, 12.12.

#### Preparation of [PtCl_3_(trz)(trzH)] (**2**)

To a solution of **1** (198 mg, 0.28 mmol) in CH_2_Cl_2_ (25 mL) was added PhICl_2_ (94 mg, 0.34 mmol), and the mixture was stirred at room temperature for 1 h. Partial evaporation of the resulting solution under reduced pressure (2 mL) and addition of Et_2_O (10 mL) led to the precipitation of a white solid, which was collected by filtration and vacuum-dried to give **2**. Yield: 200 mg (96%). ^1^H NMR (600 MHz, CD_2_Cl_2_): δ = 7.81–7.79 (m, 2H, CH), 7.65 (dd, *J*_HH_ = 7.8, 1.5 Hz, 1H, CH), 7.48–7.44 (m, 1H, CH), 7.39–7.35 (m, 2H, CH), 7.19 (td, *J*_HH_ = 7.6, 1.2 Hz, 1H, CH), 7.14 (td, *J*_HH_ = 7.6, 1.5 Hz, 1H, CH), 6.99 (dd with satellites, *J*_HH_ = 7.8, 1.3 Hz, *J*_PtH_ = 37 Hz, 1H, CH), 4.22 (s, 3H, NCH_3_), 4.10 (s, 3H, NCH_3_), 3.54 (ddd, *J*_HH_ = 14.1, 11.0, 5.3 Hz, 1H, CH_2_), 3.35 (ddd, *J*_HH_ = 14.6, 12.6, 4.5 Hz, 1H, CH_2_), 3.10 (ddd, *J*_HH_ = 14.1, 11.0, 5.4 Hz, 1H, CH_2_), 2.74 (ddd, *J*_HH_ = 14.6, 12.3, 4.3 Hz, 1H, CH_2_), 1.97–1.90 (m, 1H, CH_2_), 1.80–1.73 (m, 1H, CH_2_), 1.65–1.57 (m, 2H, CH_2_), 150–1.43 (m, 2H, CH_2_), 1.40–1.29 (m, 2H, CH_2_), 0.96 (t, *J*_HH_ = 7.4 Hz, 3H, CH_3_), 0.89 (t, *J*_HH_ = 7.4 Hz, 3H, CH_3_). ^13^C APT NMR (150 MHz, CD_2_Cl_2_): δ = 148.5 (*J*_PtC_ = 44 Hz, C), 146.0 (C), 142.1 (C), 141.0 (C), 140.2 (C), 139.4 (C), 135.1 (CH), 130.5 (CH), 130.3 (CH), 129.5 (*J*_PtC_ = 40 Hz, CH), 127.2 (CH), 126.4 (CH), 123.9 (C), 116.4 (CH), 37.2 (NCH_3_), 36.9 (NCH_3_), 32.0 (CH_2_), 31.7 (CH_2_), 27.1 (CH_2_), 23.7 (CH_2_), 23.2 (CH_2_), 23.1 (CH_2_), 14.13 (CH_3_), 14.06 (CH_3_). Anal. Calcd for C_26_H_33_Cl_3_N_6_Pt: C, 42.72; H, 4.55; N, 11.50. Found: C, 42.66, H, 4.47, N, 11.60.

#### Preparation of [PtCl_2_(trz)_2_] (**3**)

A Carius tube was charged with complex **2** (75 mg, 0.10 mmol), Na_2_CO_3_ (54 mg, 0.51 mmol), and 1,2-dichlorobenzene (3 mL), and the mixture was stirred at 120 °C for 14 h. After cooling down to room temperature, Et_2_O (10 mL) was added, and the precipitate was collected by filtration. The product was extracted with CH_2_Cl_2_ (5 × 5 mL). Partial evaporation of the combined extracts under reduced pressure (2 mL) and addition of Et_2_O (10 mL) led to the precipitation of a white solid, which was collected by filtration and vacuum-dried to give **3**. Yield: 56 mg (78%). ^1^H NMR (600 MHz, CD_2_Cl_2_): δ = 7.62 (dd with satellites, *J*_HH_ = 7.9, 1.4 Hz, *J*_PtH_ = 9 Hz, 2H, CH), 7.09 (ddd, *J*_HH_ = 7.8, 7.5, 1.2 Hz, 2H, CH), 6.86 (td, *J*_HH_ = 7.7, 1.4 Hz, 2H, CH), 6.49 (dd with satellites, *J*_HH_ = 7.8, 1.2 Hz, *J*_PtH_ = 52 Hz, 2H, CH), 4.25 (s, 6H, NCH_3_), 3.48 (ddd, *J*_HH_ = 14.2, 10.1, 5.8 Hz, 2H, CH_2_), 3.24 (ddd, *J*_HH_ = 14.3, 10.2, 6.1 Hz, 2H, CH_2_), 1.86–1.74 (m, 4H, CH_2_), 1.50 (sext, *J*_HH_ = 7.4 Hz, 4H, CH_2_), 0.97 (t, *J*_HH_ = 7.4 Hz, 6H, CH_3_). ^13^C APT NMR (150 MHz, CD_2_Cl_2_): δ = 149.3 (*J*_PtC_ = 802 Hz, C), 146.8 (*J*_PtC_ = 58 Hz, C), 142.2 (C), 133.4 (*J*_PtC_ = 25 Hz, CH), 129.8 (*J*_PtC_ = 54 Hz, CH), 126.1 (*J*_PtC_ = 814 Hz, C), 125.8 (CH), 116.1 (*J*_PtC_ = 30 Hz, CH), 37.0 (NCH_3_), 32.0 (CH_2_), 24.0 (CH_2_), 23.2 (CH_2_), 14.1 (CH_3_). Anal. Calcd for C_26_H_32_Cl_2_N_6_Pt: C, 44.96; H, 4.64; N, 12.10. Found: C, 45.03; H, 4.74; N, 12.09.

#### General Procedure for the Preparation of [Pt(trz)_2_(C∧N)]OTf (**4**)

A Carius tube was charged with complex **3** (60 mg, 0.09 mmol), AgOTf (54 mg, 0.21 mmol), the N∧CH ligand (0.45 mmol), and 1,2-dichlorobenzene (2 mL), and the mixture was stirred at 120 °C for 14 h. After cooling down to room temperature, CH_2_Cl_2_ (10 mL) was added, and the mixture was filtered through Celite. An excess of NaOAc was then added, and the suspension was stirred for 30 min and filtered through Celite. Partial evaporation of the filtrate and addition to Et_2_O (10 mL) led to the precipitation of a white solid, which was collected by filtration and vacuum-dried to give the corresponding complex **4**.

#### Data for [Pt(trz)_2_(dfppy)]OTf (**4a**)

Yield: 61 mg (73%). ^1^H NMR (600 MHz, CD_2_Cl_2_): δ = 8.45 (ddd, *J* = 9.0, 2.9, 1.4 Hz, 1H, CH), 8.05–8.00 (m, 2H, CH), 7.82 (dd, *J* = 8.0, 1.3 Hz, 1H, CH), 7.75 (dd with satellites, *J* = 8.0, 1.4 Hz, *J*_PtH_ ∼ 8 Hz, 1H, CH), 7.31 (ddd, *J* = 7.9, 7.5, 1.3 Hz, 1H, CH), 7.27 (ddd, *J* = 7.9, 7.5, 1.2 Hz, 1H, CH), 7.17–7.13 (m, 2H, CH), 7.04 (td with satellites, *J* = 7.6, 1.4 Hz, *J*_PtH_ ∼ 7 Hz, 1H, CH), 6.87 (dd with satellites, *J* = 7.4, 1.3 Hz, *J*_PtH_ = 26 Hz, 1H, CH), 6.72 (ddd, *J* = 12.7, 8.8, 2.4 Hz, 1H, CH), 6.57 (dd with satellites, *J* = 7.8, 1.2 Hz, *J*_PtH_ = 49 Hz, 1H, CH), 6.29 (dd with satellites, *J* = 7.3, 2.4 Hz, *J*_PtH_ = 32 Hz, 1H, CH), 4.13 (s, 3H, NCH_3_), 4.13 (s, 3H, NCH_3_), 2.09 (ddd, *J* = 14.8, 11.8, 4.6 Hz, 1H, CH_2_), 1.99–1.91 (m, 2H, CH_2_), 1.90–1.84 (m, 1H, CH_2_), 1.26–1.10 (m, 2H, CH_2_), 1.03–0.82 (m, 6H, CH_2_), 0.76 (t, *J*_HH_ = 7.2 Hz, 3H, CH_3_), 0.73 (t, *J*_HH_ = 7.0 Hz, 3H, CH_3_). ^13^C APT NMR (150 MHz, CD_2_Cl_2_): δ = 164.7 (dd, *J*_FC_ = 246, 10 Hz, C), 163.4 (d, *J*_FC_ = 7 Hz, C), 162.9 (dd, *J*_FC_ = 250, 11 Hz, C), 158.2 (*J*_PtC_ = 536 Hz, C), 150.4 (CH), 146.2 (C), 144.3 (C), 143.7 (C), 141.5 (C), 141.1 (CH), 140.6 (*J*_PtC_ = 508 Hz, C), 135.5 (CH), 132.8 (*J*_PtC_ = 22 Hz, CH), 131.2 (*J*_PtC_ = 29 Hz, CH), 130.8 (*J*_PtC_ = 52 Hz, CH), 128.6 (C), 126.9 (CH), 126.1 (CH), 125.2 (*J*_PtC_ = 780 Hz, C), 125.1 (*J*_FC_ = 22 Hz, CH), 124.3 (CH), 117.3 (d with satellites, *J*_FC_ = 19 Hz, *J*_PtC_ = 39 Hz, CH), 116.6 (CH), 116.5 (CH), 101.5 (t, *J*_FC_ = 27 Hz, CH), 37.3 (NCH_3_), 37.2 (NCH_3_), 32.3 (CH_2_), 32.3 (CH_2_), 24.2 (CH_2_), 24.2 (CH_2_), 23.3 (CH_2_), 23.1 (CH_2_), 13.9 (CH_3_), 13.8 (CH_3_). HRMS (ESI+) *m*/*z*: [M^+^] Calcd for C_37_H_38_F_2_N_7_Pt 813.2807; Found 813.2813. Anal. Calcd for C_38_H_38_F_5_N_7_O_3_PtS: C, 47.40; H, 3.98; N, 10.18; S, 3.33. Found: C, 47.40; H, 3.89; N, 10.20; S, 3.34.

#### Data for [Pt(trz)_2_(ppy)]OTf (**4b**)

Yield: 61 mg (76%). ^1^H NMR (600 MHz, CD_2_Cl_2_): δ = 8.11 (d, *J*_HH_ = 8.1 Hz, 1H, CH), 8.02–7.96 (m, 2H, CH), 7.88 (dd, *J*_HH_ = 7.9, 0.9 Hz, 1H, CH), 7.81 (ddd, *J*_HH_ = 7.9, 1.2, 0.5 Hz, 1H, CH), 7.74 (dd with satellites, *J*_HH_ = 8.0, 1.4 Hz, *J*_PtH_ ∼ 8 Hz, 1H, CH), 7.29 (ddd, *J*_HH_ = 8.0, 7.4, 1.3 Hz, 1H, CH), 7.25 (ddd, *J*_HH_ = 8.0, 7.4, 1.2 Hz, 1H, CH), 7.23 (ddd, *J*_HH_ = 7.9, 7.3, 1.3 Hz, 1H, CH), 7.15 (td, *J*_HH_ = 7.4, 1.2 Hz, 1H, CH), 7.11 (ddd, *J*_HH_ = 7.4, 5.7, 1.3 Hz, 1H, CH), 7.07–7.00 (m, 2H, CH), 6.92 (ddd with satellites, *J*_HH_ = 7.2, 1.3, 0.5 Hz, *J*_PtH_ = 25 Hz, 1H, CH), 6.75 (dd with satellites, *J*_HH_ = 7.5, 1.2 Hz, *J*_PtH_ = 27 Hz, 1H, CH), 6.61 (dd with satellites, *J*_HH_ = 7.8, 1.2 Hz, *J*_PtH_ = 50 Hz, 1H, CH), 4.10 (s, 3H, NCH_3_), 4.09 (s, 3H, NCH_3_), 2.03–1.95 (m, 2H, CH_2_), 1.85–1.76 (m, 2H, CH_2_), 1.18–1.10 (m, 2H, CH_2_), 1.01–0.79 (m, 6H, CH_2_), 0.73 (t, *J*_HH_ = 7.2 Hz, 3H, CH_3_), 0.71 (t, *J*_HH_ = 7.1 Hz, 3H, CH_3_). ^13^C APT NMR (150 MHz, CD_2_Cl_2_): δ = 167.1 (*J*_PtC_ = 29 Hz, C), 152.0 (*J*_PtC_ = 533 Hz, C), 150.1 (CH), 146.3 (*J*_PtC_ = 77 Hz, C), 144.9 (*J*_PtC_ = 795 Hz, C), 144.6 (C), 144.5 (C), 144.0 (*J*_PtC_ ∼ 60 Hz, C), 142.5 (*J*_PtC_ ∼ 495 Hz, C), 141.6 (C), 140.5(CH), 135.8 (*J*_PtC_ = 15 Hz, CH), 135.1 (*J*_PtC_ = 42 Hz, CH), 133.0 (*J*_PtC_ = 24 Hz, CH), 131.7 (*J*_PtC_ = 35 Hz, CH), 130.9 (*J*_PtC_ = 28 Hz, CH), 130.5 (*J*_PtC_ = 53 Hz, CH), 126.6 (CH), 126.0 (*J*_PtC_ = 794 Hz, C), 125.9 (CH), 125.54 (CH), 125.49 (CH), 124.2 (CH), 121.2 (CH), 116.4 (CH), 37.1 (NCH_3_), 37.0 (NCH_3_), 32.3 (CH_2_), 32.2 (CH_2_), 24.2 (CH_2_), 24.0 (CH_2_), 23.13 (CH_2_), 23.07 (CH_2_), 13.9 (2 CH_3_). HRMS (ESI+) *m*/*z*: [M^+^] Calcd for C_37_H_40_N_7_Pt 777.2996; Found: 777.3005. Anal. Calcd for C_38_H_40_F_3_N_7_O_3_PtS: C, 49.24; H, 4.35; N, 10.58; S, 3.46. Found: C, 49.30; H, 4.32; N, 10.50; S, 3.40.

#### Data for [Pt(trz)_2_(tpy)]OTf (**4c**)

Yield: 65 mg (80%). ^1^H NMR (600 MHz, CD_2_Cl_2_): δ = 8.04 (d, *J*_HH_ = 8.2 Hz, 1H, CH), 7.97–7.91 (m, 2H, CH), 7.80 (d, *J*_HH_ = 8.0 Hz, 1H, CH), 7.77 (d, *J*_HH_ = 8.0 Hz, 1H, CH), 7.74 (dd with satellites, *J*_HH_ = 8.0, 1.4 Hz, *J*_PtH_ ∼ 8 Hz, 1H, CH), 7.29 (td, *J*_HH_ = 7.7, 1.1 Hz, 1H, CH), 7.25 (td, *J*_HH_ = 7.7, 1.0 Hz, 1H, CH), 7.15 (td, *J*_HH_ = 7.4, 1.0 Hz, 1H, CH), 7.07–7.00 (m, 3H, CH), 6.92 (dd with satellites, *J*_HH_ = 7.2, 1.3 Hz, *J*_PtH_ = 25 Hz, 1H, CH), 6.60 (dd with satellites, *J*_HH_ = 7.8, 1.2 Hz, *J*_PtH_ = 50 Hz, 1H, CH), 6.54 (s with satellites, *J*_PtH_ = 27 Hz, 1H, CH), 4.10 (s, 3H, NCH_3_), 4.09 (s, 3H, NCH_3_), 2.12 (s, 3H, CH_3_), 2.06–1.95 (m, 2H, CH_2_), 1.86–1.79 (m, 1H, CH_2_), 1.78–1.72 (m, 1H, CH_2_), 1.20–1.11 (m, 2H, CH_2_), 1.02–0.84 (m, 6H, CH_2_), 0.73 (t, *J*_HH_ = 7.2 Hz, 3H, CH_3_), 0.73 (t, *J*_HH_ = 7.1 Hz, 3H, CH_3_). ^13^C APT NMR (150 MHz, CD_2_Cl_2_): δ = 167.2 (C), 152.1 (*J*_PtC_ = 532 Hz, C), 150.0 (CH), 146.2 (*J*_PtC_ = 76 Hz, C), 145.0 (C), 144.5 (C), 144.1 (C), 144.0 (C), 142.8 (C), 142.2 (C), 142.0 (C), 141.6 (C), 140.3 (CH), 135.9 (CH), 135.8 (CH), 132.9 (*J*_PtC_ = 24 Hz, CH), 130.9 (*J*_PtC_ = 28 Hz, CH), 130.5 (*J*_PtC_ = 52 Hz, CH), 126.5 (CH), 126.4 (CH), 126.0 (*J*_PtC_ = 793 Hz, C), 125.8 (*J*_PtC_ = 19 Hz, CH), 125.5 (CH), 123.6 (CH), 120.9 (CH), 116.4 (2 CH), 37.1 (NCH_3_), 37.0 (NCH_3_), 32.3 (CH_2_), 32.2 (CH_2_), 24.2 (CH_2_), 24.0 (CH_2_), 23.2 (2 CH_2_), 21.9 (CH_3_), 14.0 (CH_3_), 13.9 (CH_3_). HRMS (ESI+) *m*/*z*: [M^+^] Calcd for C_38_H_43_N_7_Pt 791.3152; Found 791.3153. Anal. Calcd for C_39_H_42_F_3_N_7_O_3_PtS: C, 49.78; H, 4.50; N, 10.42; S, 3.41. Found: C, 49.60; H, 4.60; N, 10.24; S, 2.90.

#### Data for [Pt(trz)_2_(thpy)]OTf (**4d**)

Yield: 59 mg (73%). ^1^H NMR (600 MHz, CD_2_Cl_2_): δ = 7.88 (td, *J*_HH_ = 7.7, 1.4 Hz, 1H, CH), 7.82–7.78 (m, 2H, CH), 7.72 (dd with satellites, *J*_HH_ = 7.9, 1.4 Hz, *J*_PtH_ ∼ 8 Hz, 1H, CH), 7.70 (dt, *J*_HH_ = 8.1, 1.1 Hz, 1H, CH), 7.39 (d with satellites, *J*_HH_ = 4.7 Hz, *J*_PtH_ ∼ 5 Hz, 1H, CH), 7.30 (ddd, *J*_HH_ = 7.9, 7.5, 1.3 Hz, 1H, CH), 7.23 (ddd, *J*_HH_ = 8.0, 7.5, 1.2 Hz, 1H, CH), 7.14 (td, *J*_HH_ = 7.4, 1.2 Hz, 1H, CH), 7.00 (td with satellites, *J*_HH_ = 7.7, 1.4 Hz, *J*_PtH_ ∼ 8 Hz, 1H, CH), 6.93 (ddd, *J*_HH_ = 7.2, 5.6, 1.3 Hz, 1H, CH), 6.90 (dd with satellites, *J*_HH_ = 7.3, 1.3 Hz, *J*_PtH_ = 28 Hz, 1H, CH), 6.63 (d with satellites, *J*_HH_ = 7.9 Hz, *J*_PtH_ = 52 Hz, 1H, CH), 6.43 (d with satellites, *J*_HH_ = 4.7 Hz, *J*_PtH_ = 10 Hz, 1H, CH), 4.13 (s, 3H, NCH_3_), 4.12 (s, 3H, NCH_3_), 2.12 (ddd, *J*_HH_ = 14.3, 11.7, 5.1 Hz, 1H, CH_2_), 2.06–1.91 (m, 3H, CH_2_), 1.24–1.15 (m, 2H, CH_2_), 1.10–0.84 (m, 6H, CH_2_), 0.78 (t, *J*_HH_ = 7.2 Hz, 3H, CH_3_), 0.74 (t, *J*_HH_ = 7.2 Hz, 3H, CH_3_). ^13^C APT NMR (150 MHz, CD_2_Cl_2_): δ = 162.3 (C), 156.6 (*J*_PtC_ = 544 Hz, C), 150.0 (CH), 146.1 (*J*_PtC_ = 74 Hz, C), 144.5 (C), 144.3 (C), 144.2 (C), 144.1 (C), 141.9 (*J*_PtC_ = 46 Hz, C), 141.4 (C), 140.8 (CH), 140.3 (*J*_PtC_ = 530 Hz, C), 135.7 (*J*_PtC_ = 17 Hz, CH), 133.8 (*J*_PtC_ = 60 Hz, CH), 133.5 (*J*_PtC_ = 27 Hz, CH), 130.9 (*J*_PtC_ = 30 Hz, CH), 130.8 (*J*_PtC_ = 36 Hz, CH), 130.2 (*J*_PtC_ = 53 Hz, CH), 126.7 (CH), 125.7 (CH), 122.2 (*J*_PtC_ = 785 Hz, C), 121.8 (CH), 120.2 (CH), 116.4 (*J*_PtC_ = 19 Hz, CH), 116.2 (*J*_PtC_ = 30 Hz, CH), 37.2 (NCH_3_), 37.1 (NCH_3_), 32.3 (CH_2_), 32.2 CH_2_), 24.3 (CH_2_), 24.2 (CH_2_), 23.3 (CH_2_), 23.2 (CH_2_), 14.0 (2 CH_3_). HRMS (ESI+) *m*/*z*: [M^+^] Calcd for C_35_H_38_N_7_PtS 783.2559; Found 783.2550. Anal. Calcd for C_36_H_38_F_3_N_7_O_3_PtS_2_: C, 46.35; H, 4.11; N, 10.51; S, 6.87. Found: C, 46.19; H, 4.12; N, 10.30; S, 6.79.

#### Data for [Pt(trz)_2_(flpy)]OTf (**4e**)

Yield: 65 mg (72%). ^1^H NMR (600 MHz, CD_2_Cl_2_): δ = 8.19 (d, *J*_HH_ = 8.2 Hz, 1H, CH), 8.03––7.97 (m, 2H, CH), 7.96 (s, 1H, CH), 7.83–7.81 (m, 1H, CH), 7.78 (dd with satellites, *J*_HH_ = 8.0, 1.3 Hz, *J*_PtH_ ∼ 8 Hz, 1H, CH), 7.43 (dt, *J*_HH_ = 7.4, 0.9 Hz, 1H, CH), 7.33–7.27 (m, 4H, CH), 7.20 (td, *J*_HH_ = 7.4, 1.1 Hz, 1H, CH), 7.16 (td, *J*_HH_ = 7.4, 1.2 Hz, 1H, CH), 7.11–7.06 (m, 2H, CH), 7.04 (s with satellites, *J*_PtH_ = 28 Hz, 1H, CH), 6.95 (ddd with satellites, *J*_HH_ = 7.3, 1.3, 0.5 Hz, *J*_PtH_ = 25 Hz, 1H, CH), 6.67 (dd with satellites, *J*_HH_ = 7.8, 1.2 Hz, *J*_PtH_ = 50 Hz, 1H, CH), 4.08 (s, 6H, NCH_3_), 2.07–1.95 (m, 2H, CH_2_), 1.93–1.80 (m, 2H, CH_2_), 1.53 (s, 3H, CH_3_), 1.48 (s, 3H, CH_3_), 1.17–1.09 (m, 2H, CH_2_), 1.02–0.80 (m, 4H, CH_2_), 0.72 (t, *J*_HH_ = 7.1 Hz, 3H, CH_3_), 0.67–0.58 (m, 2H, CH_2_), 0.48 (t, *J*_HH_ = 7.3 Hz, 3H, CH_3_). ^13^C APT NMR (150 MHz, CD_2_Cl_2_): δ = 167.1 (*J*_PtC_ = 29 Hz, C), 155.1 (C), 151.3 (C), 151.1 (*J*_PtC_ = 532 Hz, C), 150.2 (CH), 146.3 (C), 145.0 (C), 144.6 (C), 144.2 (C), 144.1 (C), 143.4 (C), 142.9 (*J*_PtC_ = 37 Hz, C), 142.6 (C), 141.8 (C), 140.3 (CH), 138.7 (C), 135.9 (CH), 133.0 (*J*_PtC_ = 22 Hz, CH), 131.0 (*J*_PtC_ = 28 Hz, CH), 130.6 (*J*_PtC_ = 52 Hz, CH), 128.6 (CH), 127.5 (CH), 126.5 (CH), 126.3 (*J*_PtC_ = 790 Hz, C), 126.0 (*J*_PtC_ = 42 Hz, CH), 125.7 (CH), 123.6 (CH), 123.3 (CH), 121.1 (CH), 120.7 (CH), 120.2 (*J*_PtC_ = 20 Hz, CH), 116.5 (*J*_PtC_ = 30 Hz, CH), 116.4 (*J*_PtC_ ∼ 18 Hz, CH), 47.1 (C), 37.1 (NCH_3_), 37.0 (NCH_3_), 32.4 (CH_2_), 32.3 (CH_2_), 27.7 (CH_3_), 27.6 (CH_3_), 24.3 (CH_2_), 24.2 (CH_2_), 23.2 (CH_2_), 23.1 (CH_2_), 14.0 (CH_3_), 13.8 (CH_3_). HRMS (ESI+) *m*/*z*: [M^+^] Calcd for C_46_H_48_N_7_Pt 893.3617; Found: 893.3632. Anal. Calcd for C_47_H_48_F_3_N_7_O_3_PtS: C, 54.12; H, 4.64; N, 9.40; S, 3.07. Found: C, 54.11; H, 4.55; N, 9.33; S, 2.99.

## Supporting Information Available

Photophysical characterization, X-ray structure determinations, crystallographic data, NMR spectra, electrochemical measurements, excitation and emission spectra, and computational methods and data (PDF)
