# Supplementary material for: Phosphorescent Tris-cyclometalated Pt(IV) Complexes with Mesoionic N-Heterocyclic Carbene and 2-Arylpyridine Ligands
Source: Inorg Chem. 2022 Jul 21;61(30):12033–42. doi: 10.1021/acs.inorgchem.2c02039 (PMC9377419; doi:10.1021/acs.inorgchem.2c02039)
Supplement: Supplementary file 1 — ic2c02039_si_001.pdf [file ic2c02039_si_001.pdf]

SUPPORTING INFORMATION

Phosphorescent Tris-cyclometalated Pt(IV) Complexes with Mesoionic *N*-Heterocyclic Carbene and 2-Arylpyridine Ligands

Ángela Vivancos,[†] *Delia Bautista*[‡] *and Pablo González-Herrero*[*,†]

[†]Departamento de Química Inorgánica, Facultad de Química, Universidad de Murcia, Campus de Espinardo, 19, 30100 Murcia, Spain.

[‡]Área Científica y Técnica de Investigación, Universidad de Murcia, Campus de Espinardo, 21, 30100 Murcia, Spain.

Contents:

1. Photophysical characterization 2
2. X-Ray structure determinations 2
3. NMR spectra of new compounds 3
4. Photostability tests 17
5. Excitation and emission spectra 22
6. Electrochemical measurements 26
7. Computational methods and data 27
8. References 34

1. Photophysical characterization

UV-vis absorption spectra were registered on a Perkin-Elmer Lambda 750S spectrophotometer. Excitation and emission spectra were registered on a Jobin Yvon Fluorolog 3-22 spectrofluorometer; the measurements in solution were carried out in a right-angle configuration using 10 mm quartz fluorescence cells (298 K) or 5 mm quartz NMR tubes (77 K). For low-temperature measurements, a liquid nitrogen Dewar accessory was employed. The emission data in PMMA matrix were measured in a front-face configuration, using quartz slides as sample holders. Emission lifetimes were determined using an IBH FluoroHub controller in MCS mode and the Fluorolog's FL-1040 phosphorimeter pulsed xenon lamp as excitation source; the estimated uncertainty is ±10% or better. Emission quantum yields were determined using a Hamamatsu C11347 Absolute PL Quantum Yield Spectrometer; the estimated uncertainty is ±5% or better. All emission data were registered under rigorous exclusion of oxygen.

2. X-Ray structure determinations

Single crystals of complexes **1**, **2**, **4b** and **4d** suitable for X-ray diffraction were obtained by slow diffusion of Et_2O into solutions of the complexes in CH_2Cl_2. The data were collected on a Bruker D8 QUEST diffractometer with monochromated Mo-$K\alpha$ radiation performing φ and ω scans. The structures were solved by direct methods and refined anisotropically on F^2 using the program SHELXL-2018 (G. M. Sheldrick, University of Göttingen).[1,2] Methyl hydrogens were included as part of rigid idealized methyl groups allowed to rotate but not tip; other hydrogens were included using a riding model. Numerical details are given in Table S1. *Special features of refinement:* In **4b**, the phenyl and pyridine rings of the ppy ligand could not be distinguished by the refinement model. In **4d**, the positions of the thiophene and pyridine rings are disordered (*ca.* 81:19 %).

Table S1. Crystallographic data for **1**, **2**, **4b** and **4d**.

	**1**	**2**	**4b**	**4d**
formula	$C_{26}H_{34}Cl_2N_6Pt$	$C_{26}H_{33}Cl_3N_6Pt$	$C_{38}H_{40}F_3N_7O_3PtS$	$C_{36}H_{38}F_3N_7O_3PtS_2$
fw	696.58	731.02	926.92	932.94
T (K)	100(2)	100(2)	100(2)	100(2)
λ	0.71073	0.71073	0.71073	0.71073
cryst syst	Monoclinic	Monoclinic	Orthorhombic	Orthorhombic
space group	C2/c	$P2_1/c$	Pbca	Pbca
a (Å)	14.3731(15)	18.7363(13)	17.6769(11)	17.6856(9)
b (Å)	8.5933(9)	11.8902(8)	16.7845(11)	16.5934(8)
c (Å)	22.904(2)	25.2817(16)	24.5723(15)	24.4700(12)
α (°)	90	90	90	90
β (°)	102.480(4)	103.775(2)	90	90
γ (°)	90	90	90	90
V (Å^3)	2762.0(5)	5470.2(6)	7290.6(8)	7181.1(6)
Z	4	8	8	8
ρ_{calcd} (Mg m^{-3})	1.675	1.775	1.689	1.726
μ (mm^{-1})	5.300	5.451	3.970	4.088
R1^a	0.0193	0.0214	0.0337	0.0217
wR2^b	0.0420	0.0508	0.0685	0.0424

^{a}R1 = $\Sigma||F_o|-|F_c||/\Sigma|F_o|$ for reflections with $I > 2\sigma(I)$. ^{b}wR2 = $[\Sigma[w(F_o^2-F_c^2)^2/\Sigma[w(F_o^2)^2]]^{0.5}$ for all reflections; $w^{-1} = \sigma^2(F^2) + (aP)^2 + bP$, where $P = (2F_c^2 + F_o^2)/3$ and a and b are constants set by the program.

3. NMR spectra of new compounds

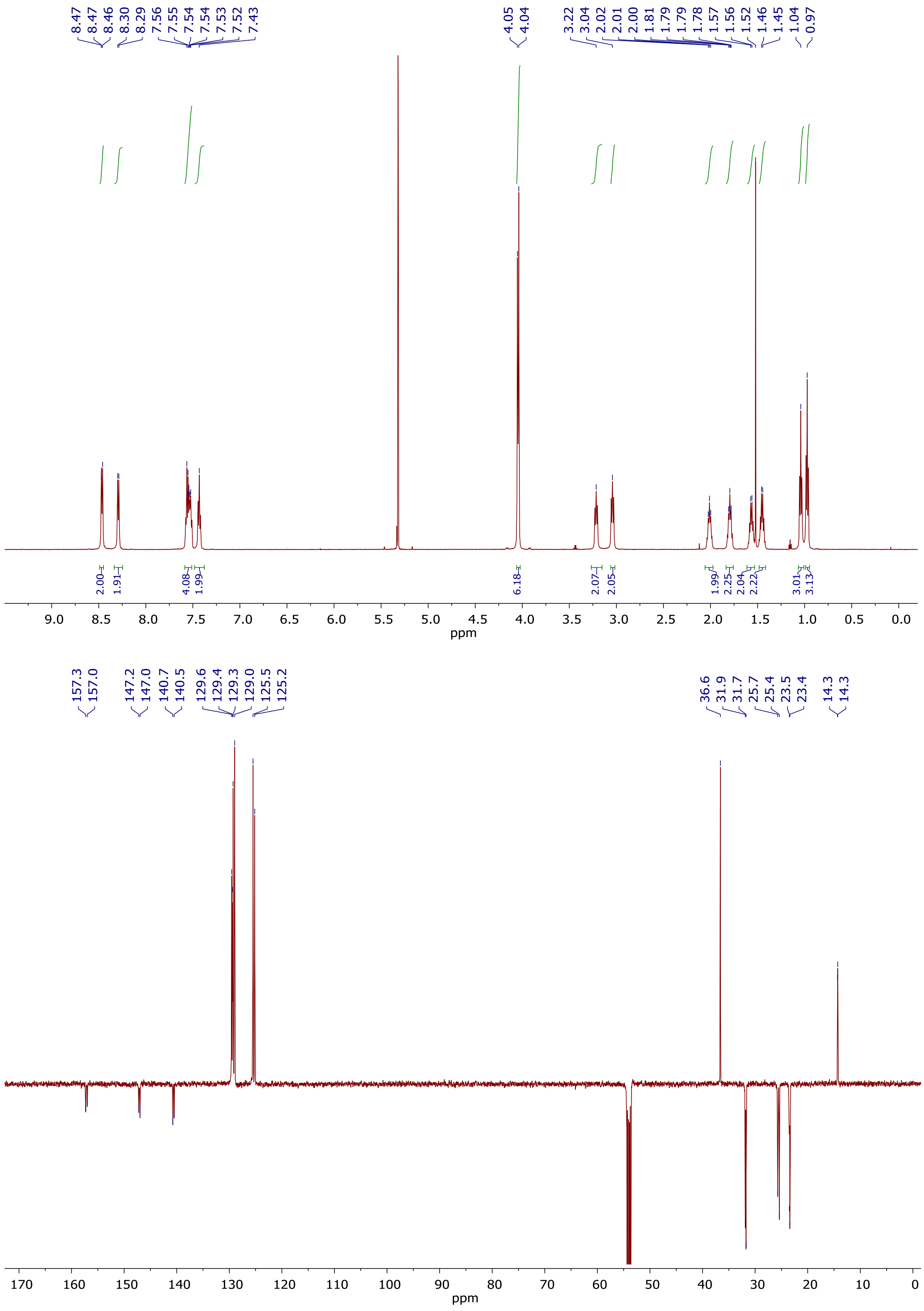

Figure S1. ^{1}H (top) and $^{13}C\{^{1}H\}$ APT (bottom) NMR spectra of complex **1** (CD_2Cl_2, 600 and 151 MHz, respectively)

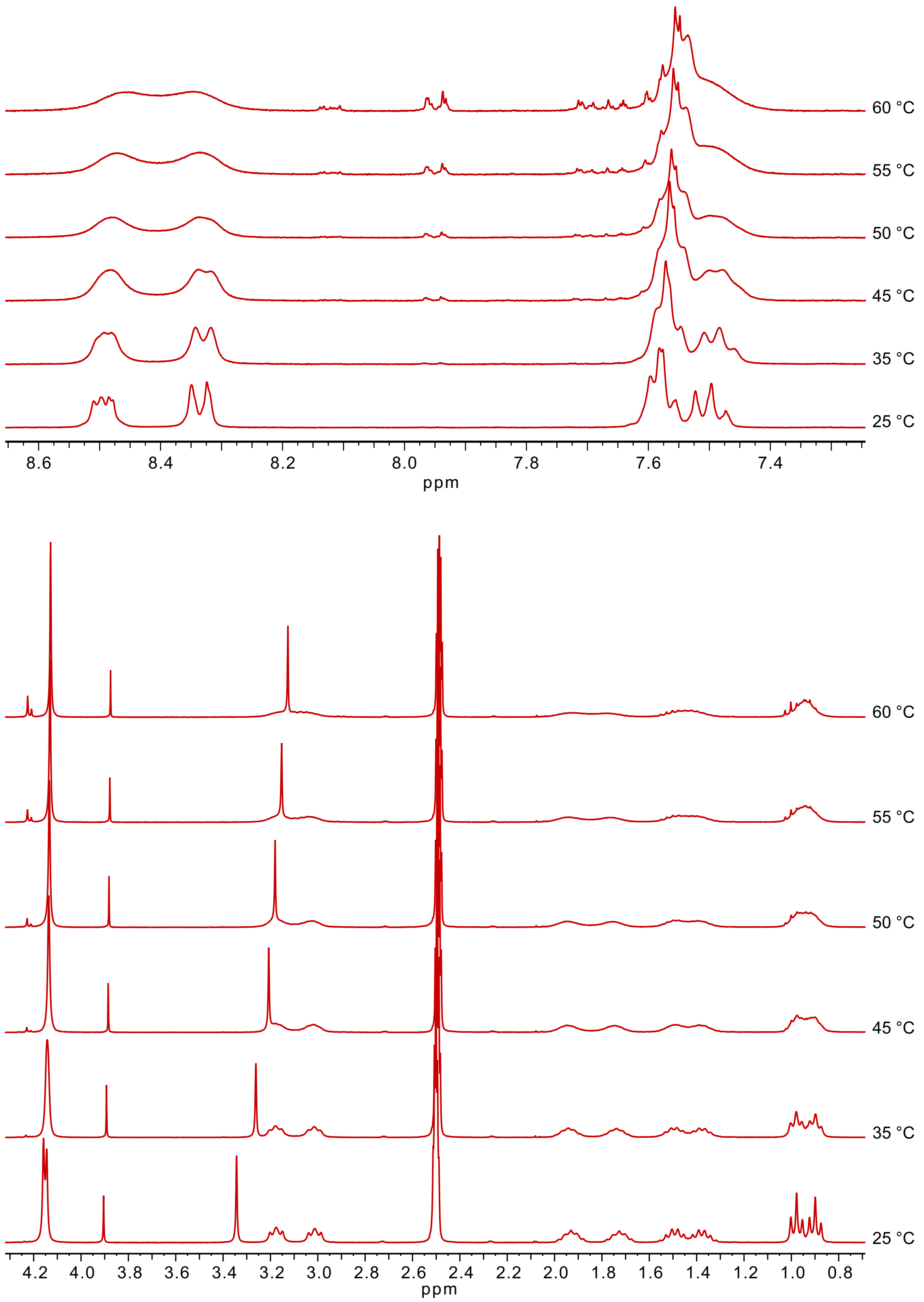

Figure S2. Variable-temperature ^{1}H NMR spectra of complex **1** (DMSO-d_6, 300 MHz). Top: aromatic region. Bottom: aliphatic region.

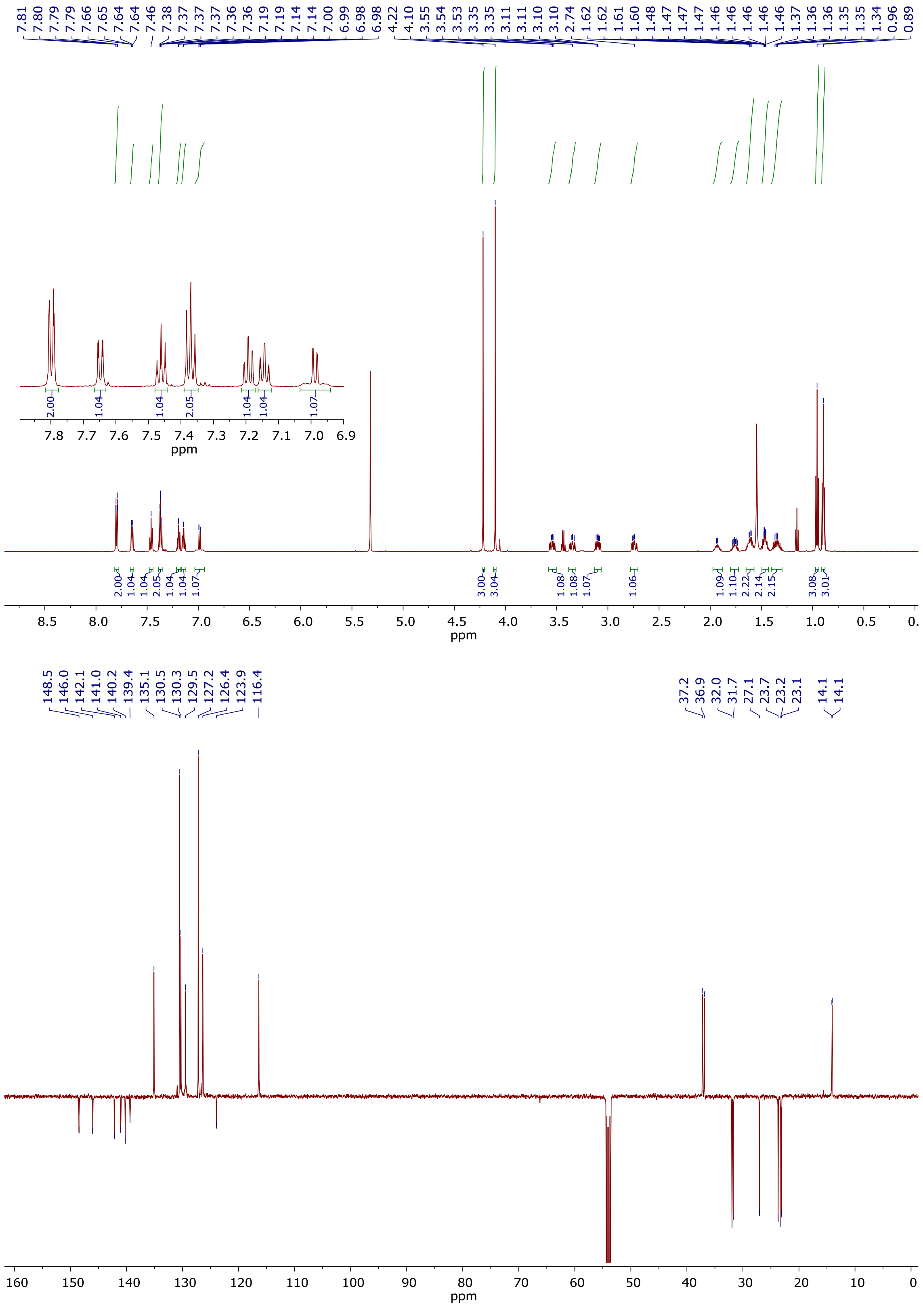

Figure S3. ^{1}H (top) and $^{13}C\{^{1}H\}$ APT (bottom) NMR spectra of complex **2** (CD_2Cl_2, 600 and 151 MHz, respectively).

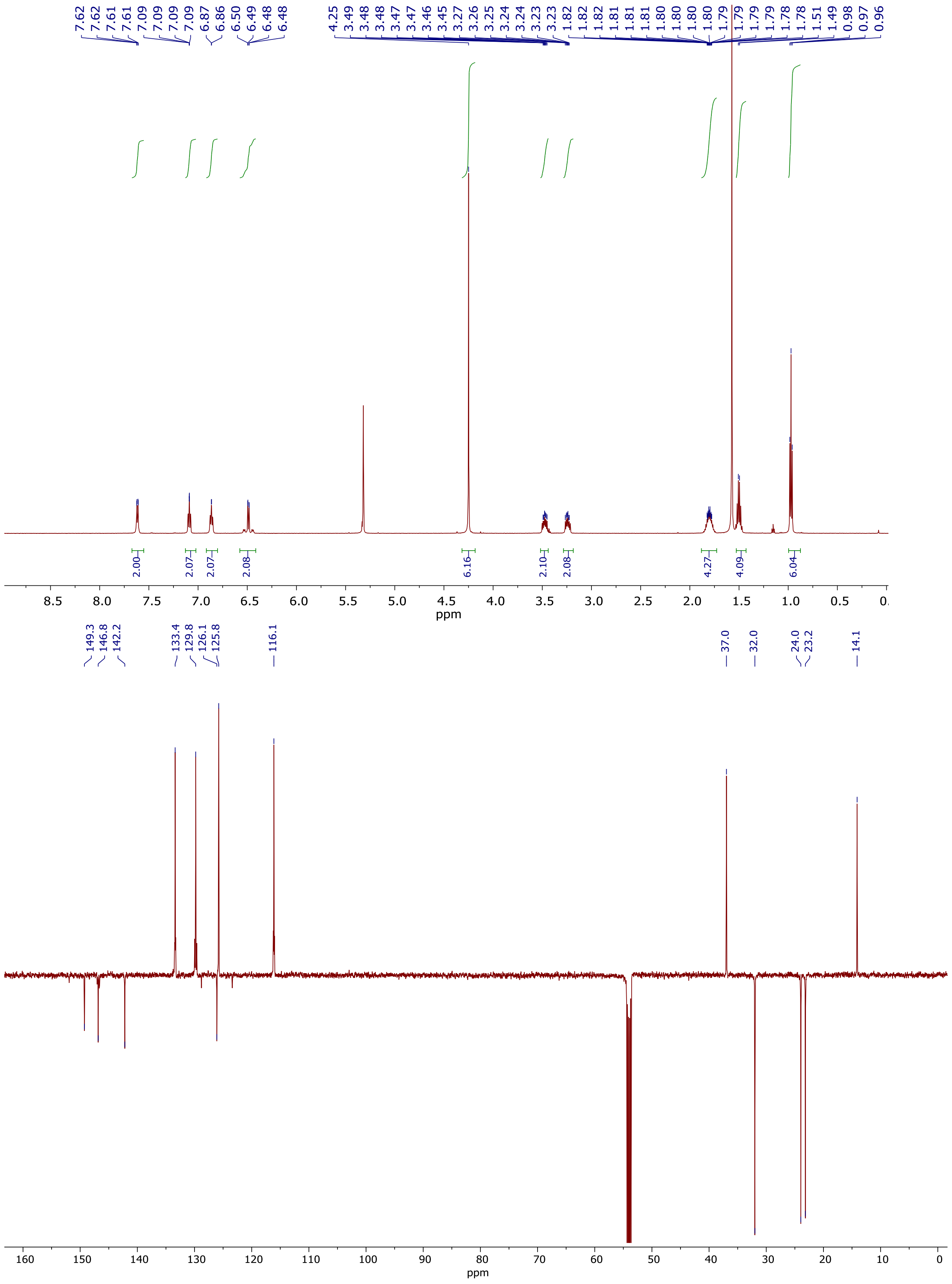

Figure S4. ^1H (top) and $^{13}C\{^1H\}$ APT (bottom) NMR spectra of complex **3** (CD_2Cl_2, 600 and 151 MHz, respectively).

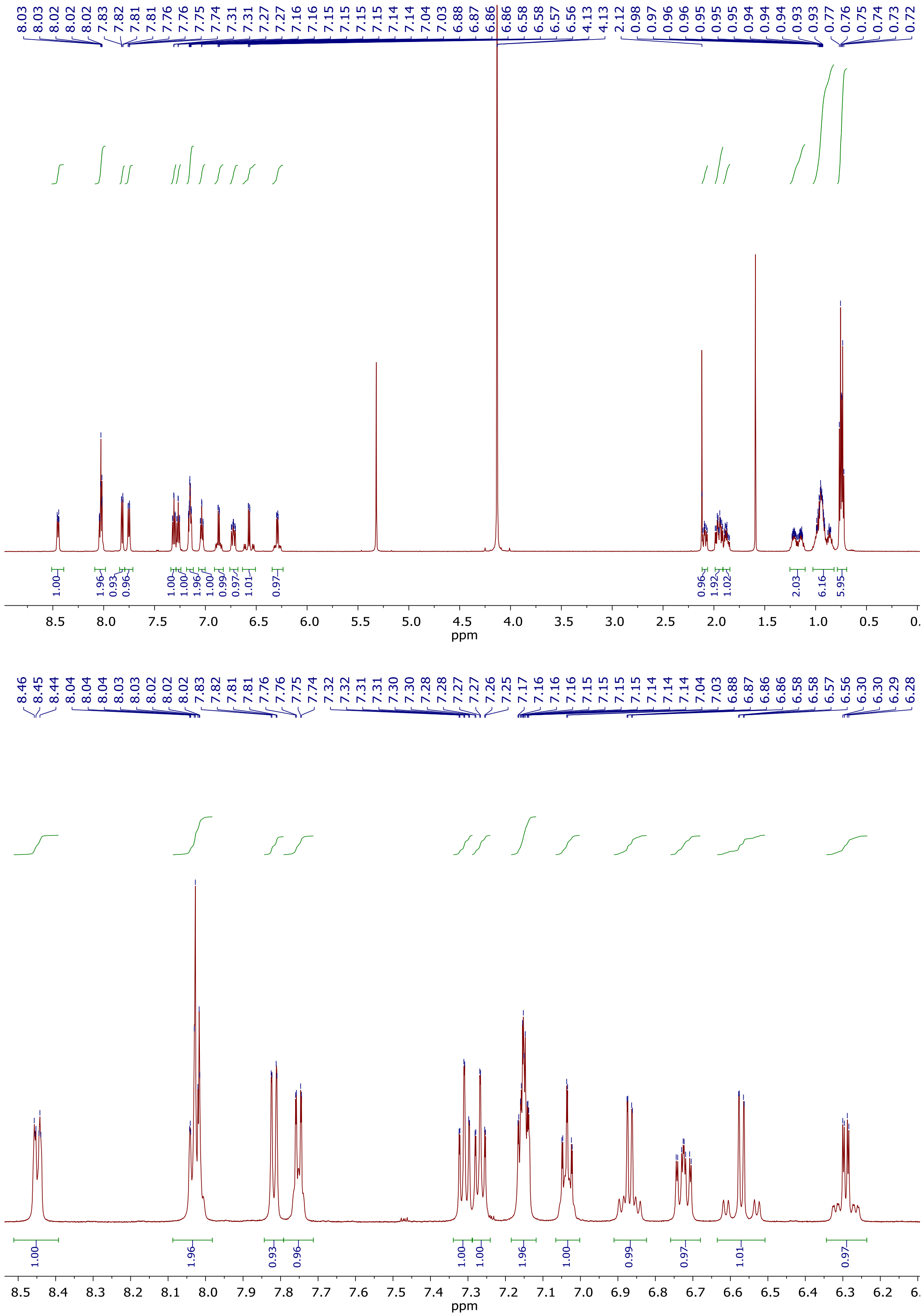

Figure S5. ^{1}H NMR spectrum of complex **4a** (CD_2Cl_2, 600 MHz) (top) and selected area (bottom).

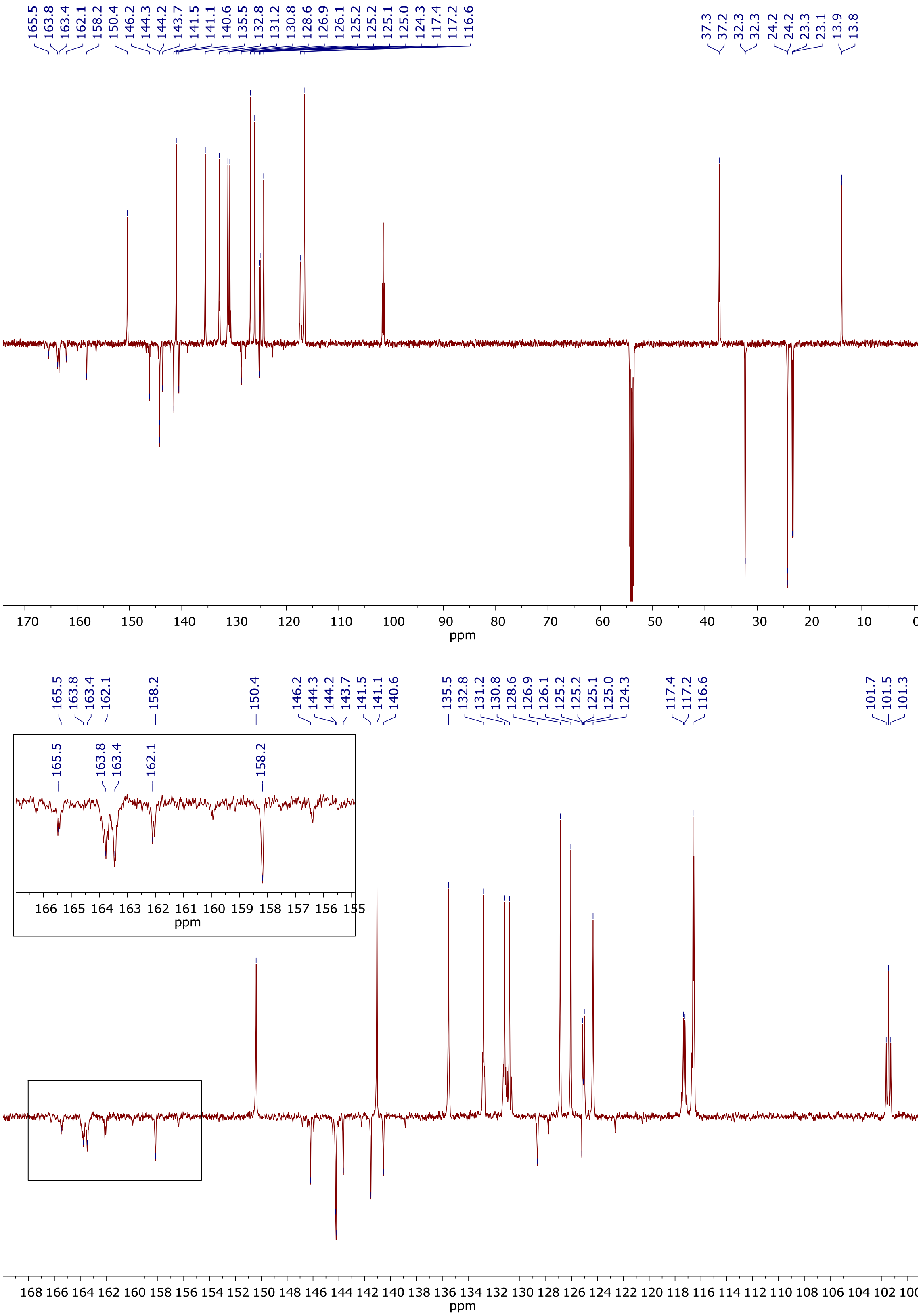

Figure S6. $^{13}C\{^{1}H\}$ APT NMR spectrum of complex **4a** (CD_2Cl_2, 151 MHz) (top) and selected area (bottom).

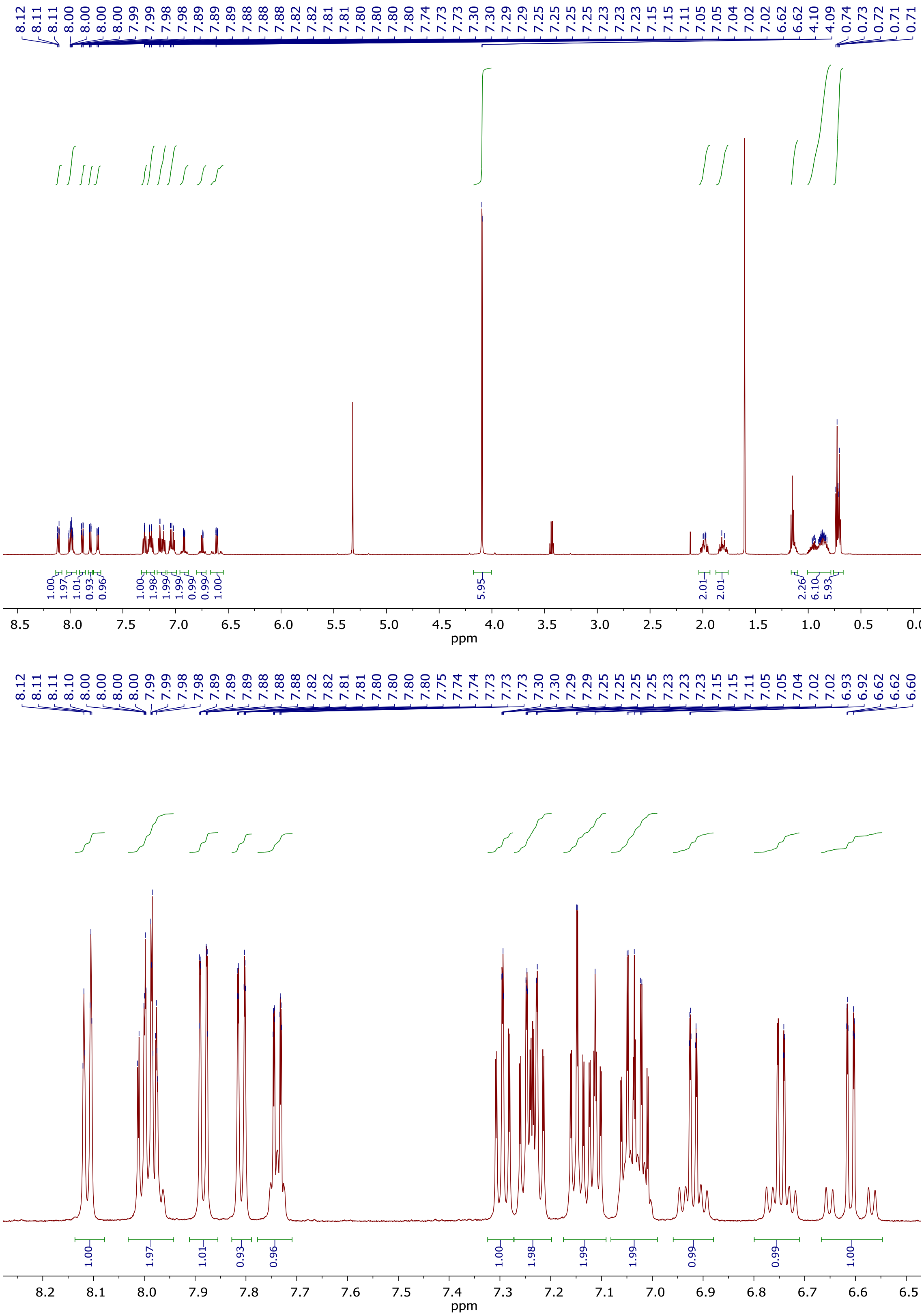

Figure S7. ^{1}H NMR spectrum of complex **4b** (CD_2Cl_2, 600 MHz) (top) and selected area (bottom).

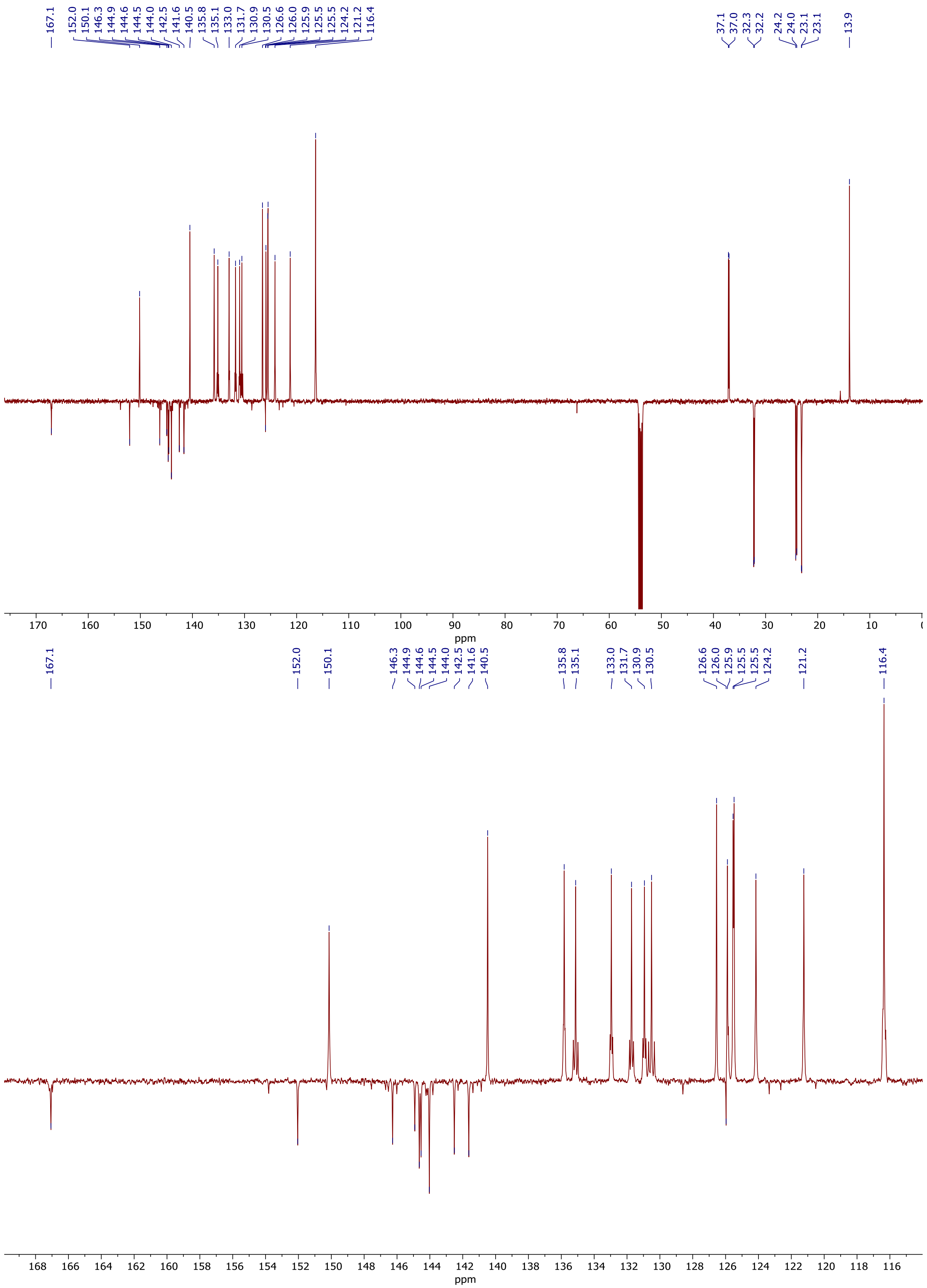

Figure S8. $^{13}C\{^{1}H\}$ APT NMR spectrum of complex **4b** (CD_2Cl_2, 151 MHz) (top) and selected area (bottom).

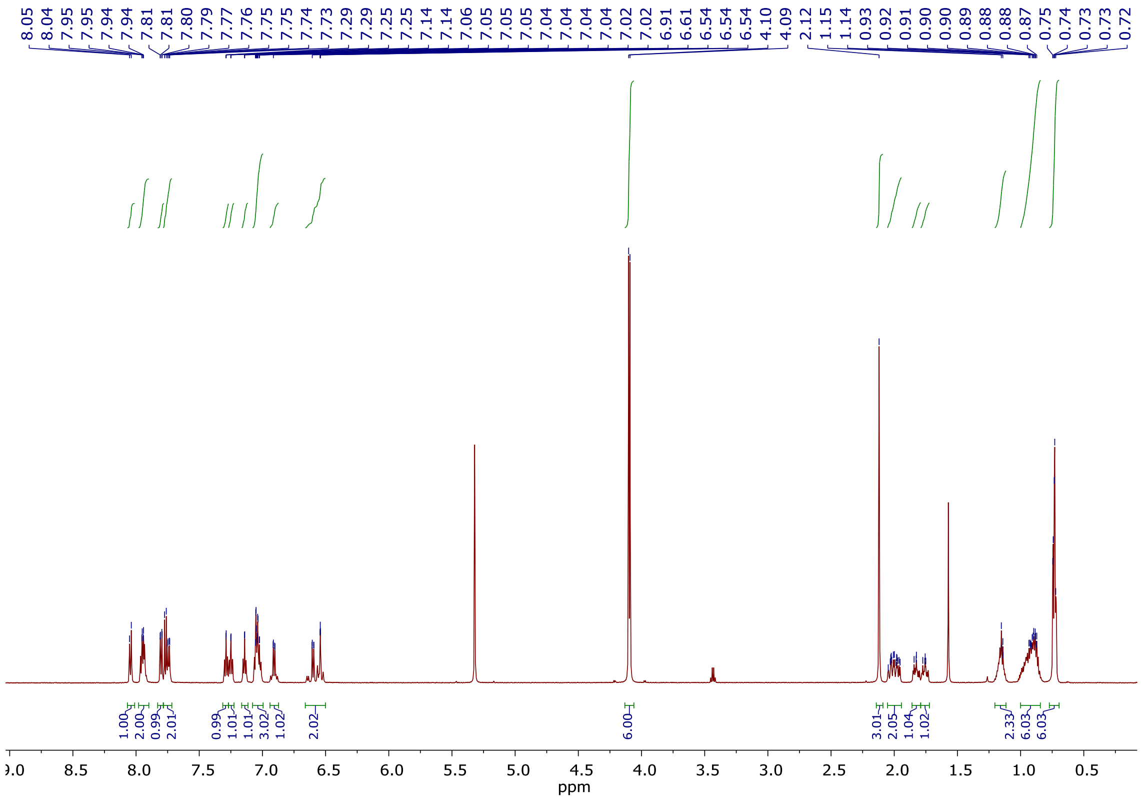

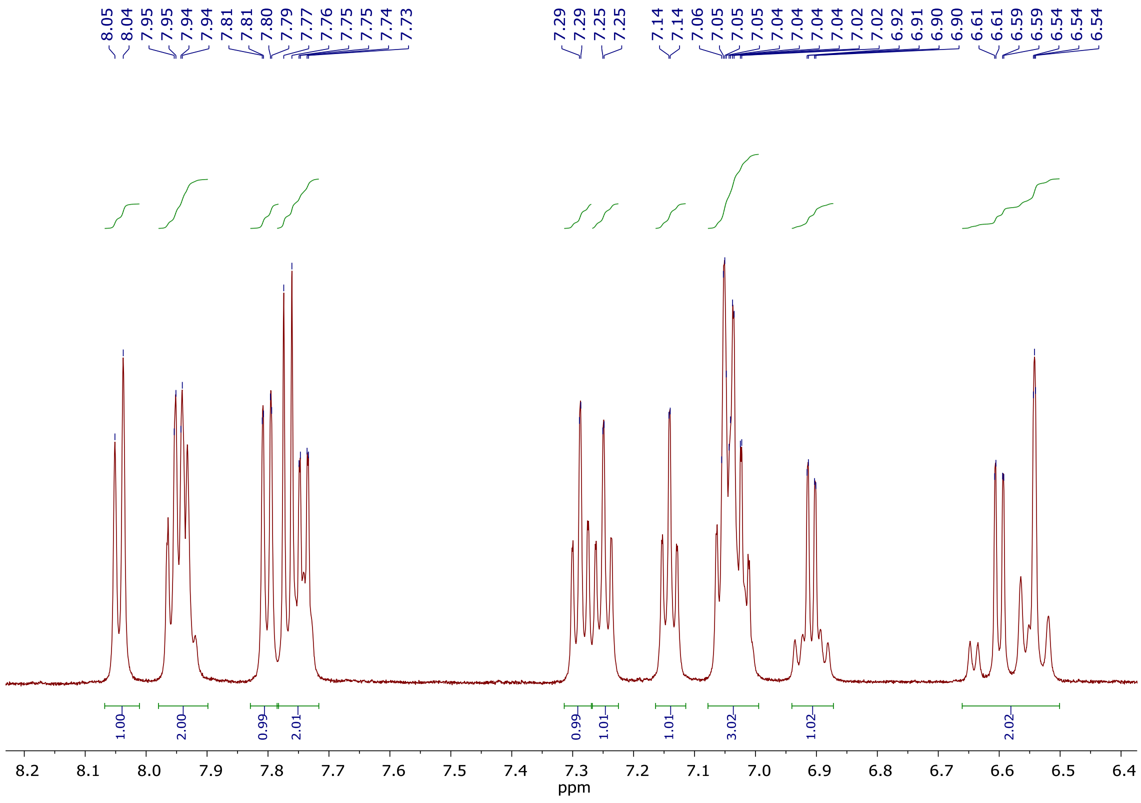

Figure S9. ^{1}H NMR spectrum of complex **4c** (CD_2Cl_2, 600 MHz) (top) and selected area (bottom).

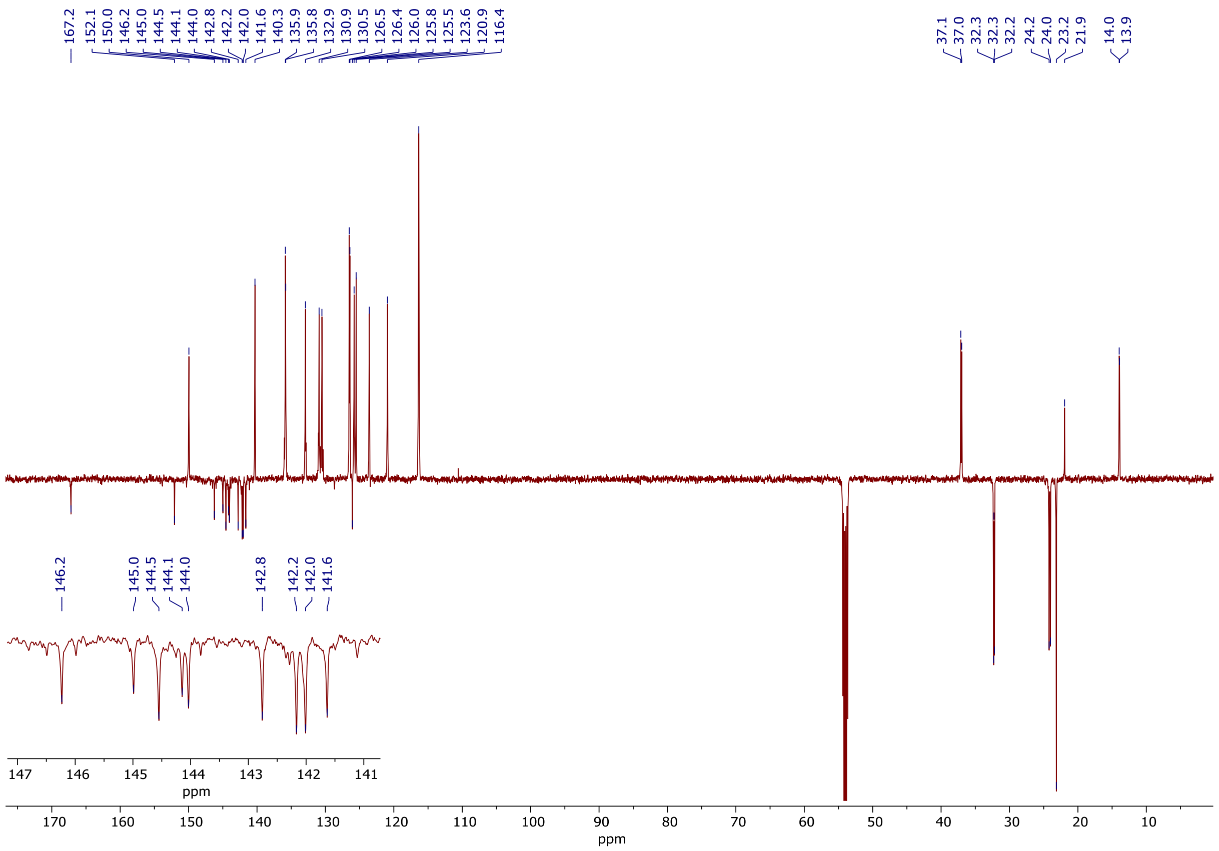

Figure S10. $^{13}C\{^{1}H\}$ APT NMR spectrum of complex **4c** (CD_2Cl_2, 151 MHz).

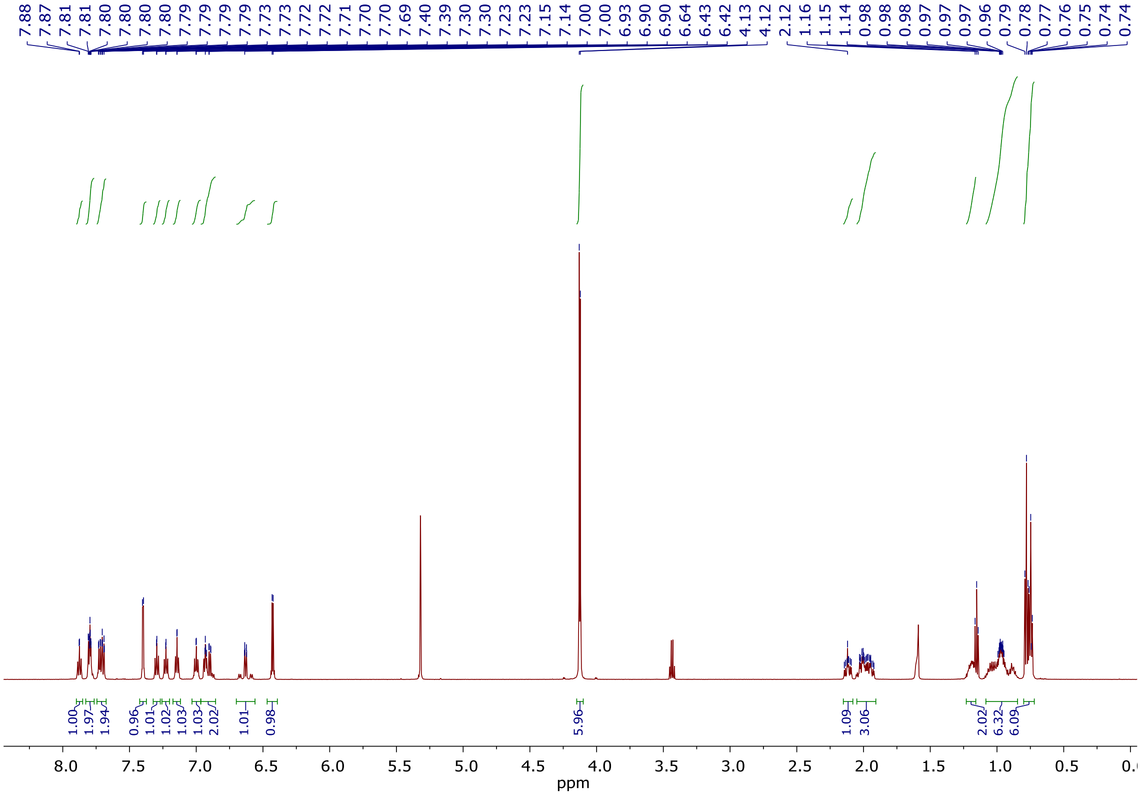

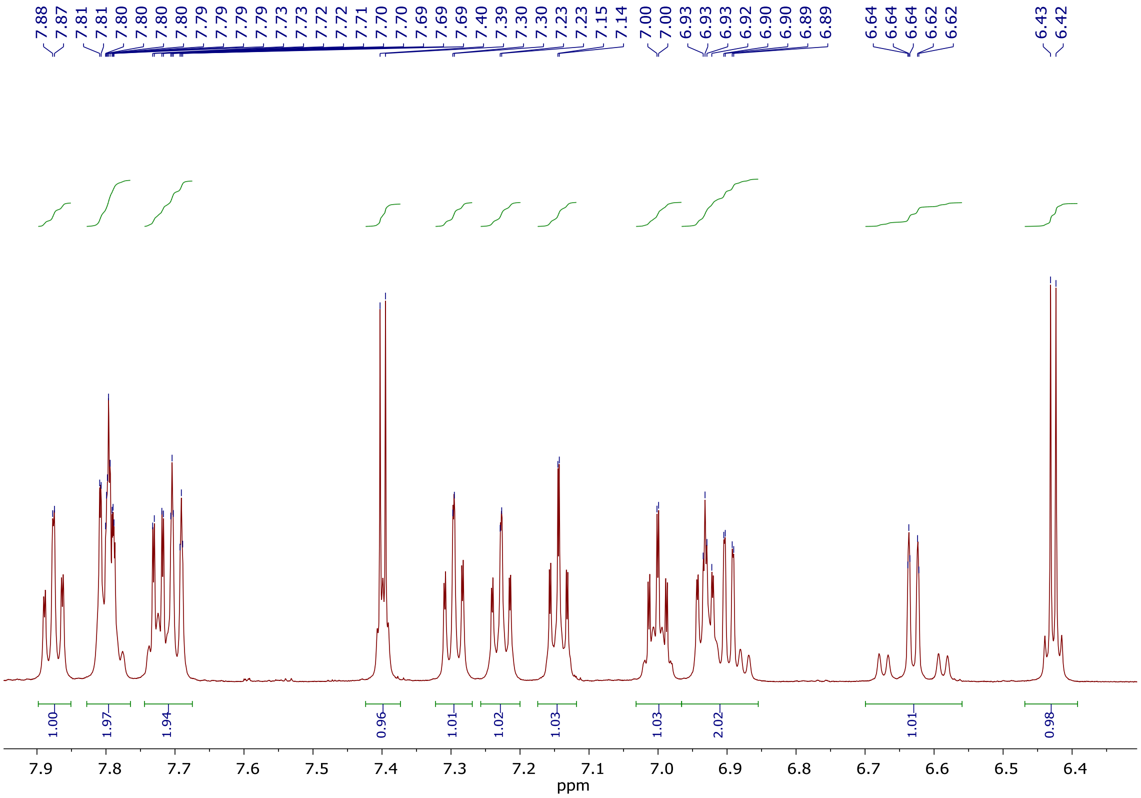

Figure S11. ^{1}H NMR spectrum of complex **4d** (CD_2Cl_2, 600 MHz) (top) and selected area (bottom).

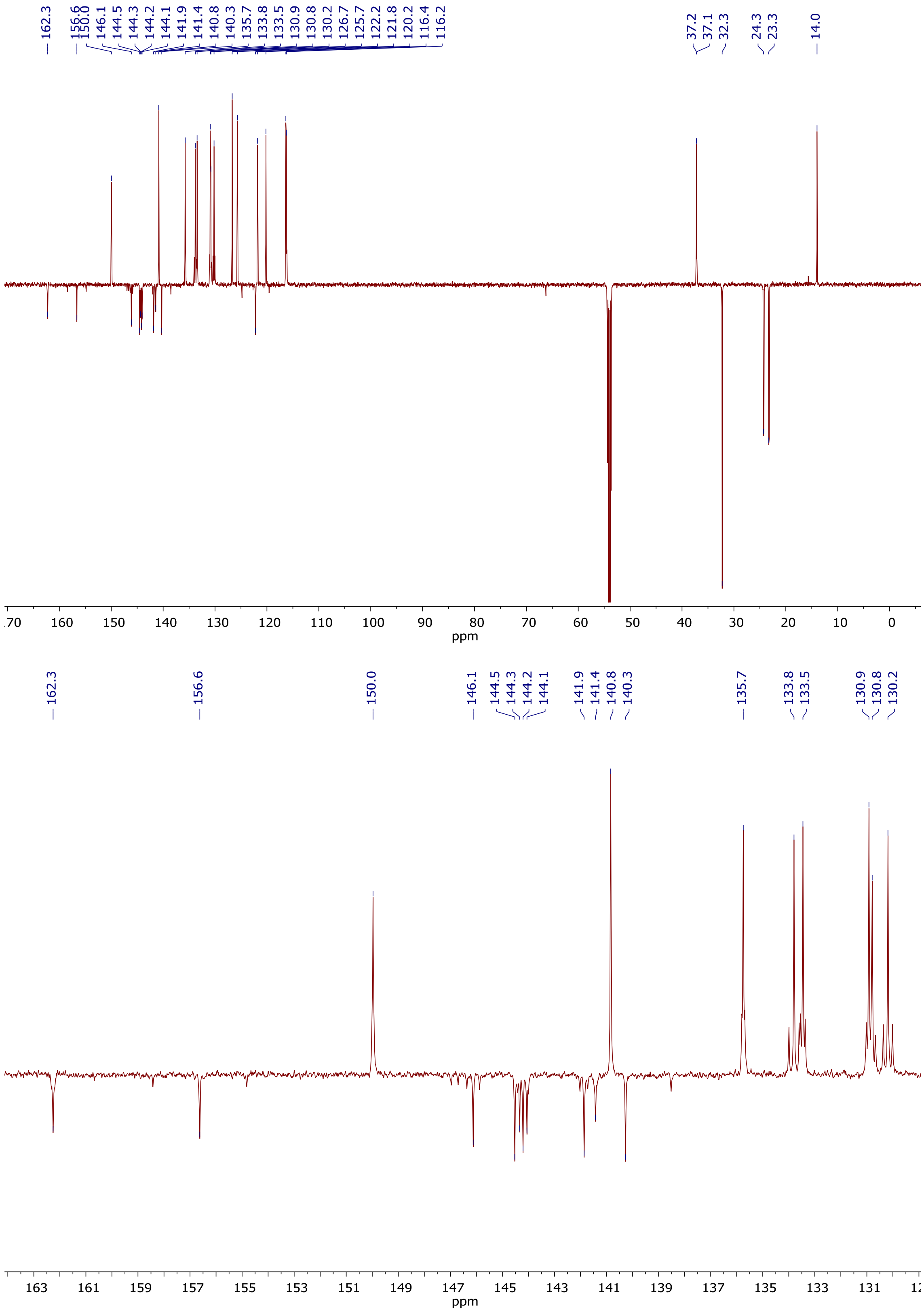

Figure S12. $^{13}C\{^{1}H\}$ APT NMR spectrum of complex **4d** (CD_2Cl_2, 151 MHz) (top) and selected area (bottom).

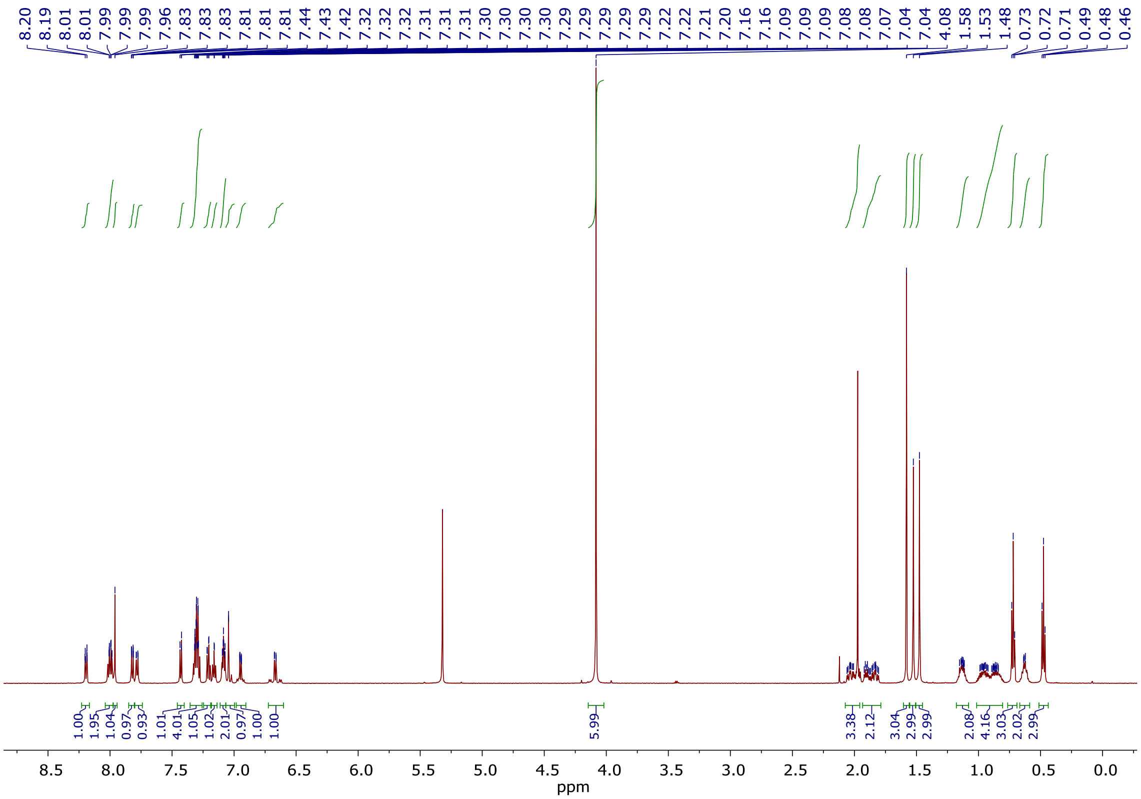

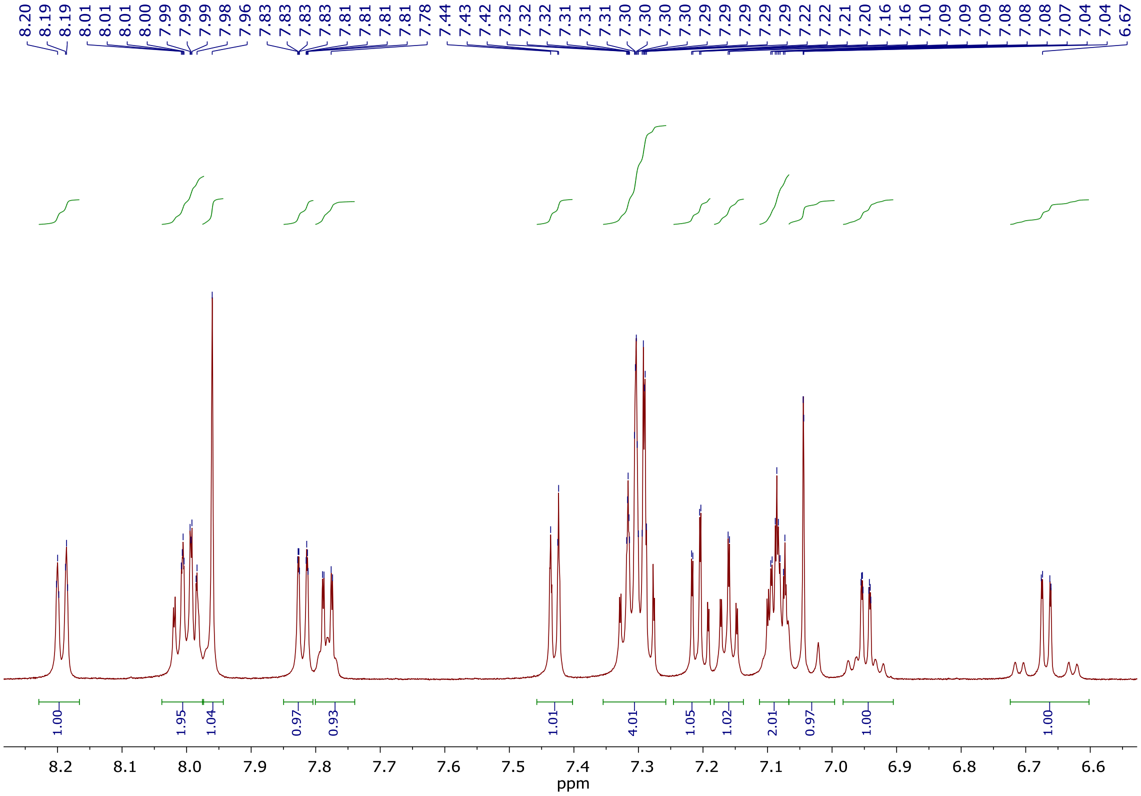

Figure S13. ^{1}H NMR spectrum of complex **4e** (CD_2Cl_2, 600 MHz) (top) and selected area (bottom).

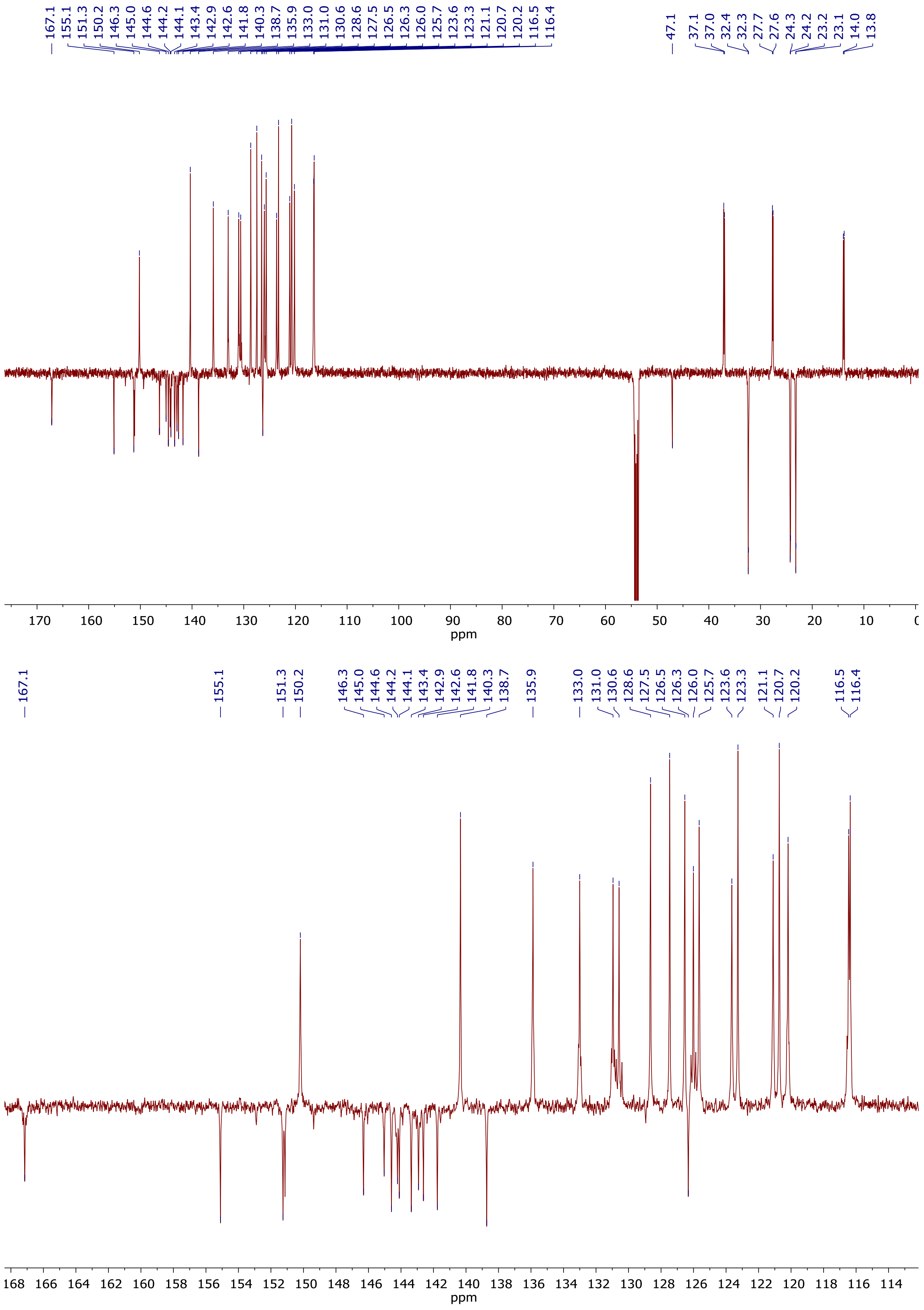

Figure S14. $^{13}C\{^{1}H\}$ APT NMR spectrum of complex **4e** (CD_2Cl_2, 151 MHz) (top) and selected area (bottom).

4. Photostability tests

Photostability tests were carried out for **4a-e** by irradiating degassed CD_3CN solutions of the complexes in quartz NMR tubes with a 36 W Philips UVB Narrowband lamp centered at 310 nm. ^1H NMR spectra were registered after 3 and 6 hours of irradiation and are shown below. Cyclohexane was employed as an internal standard.

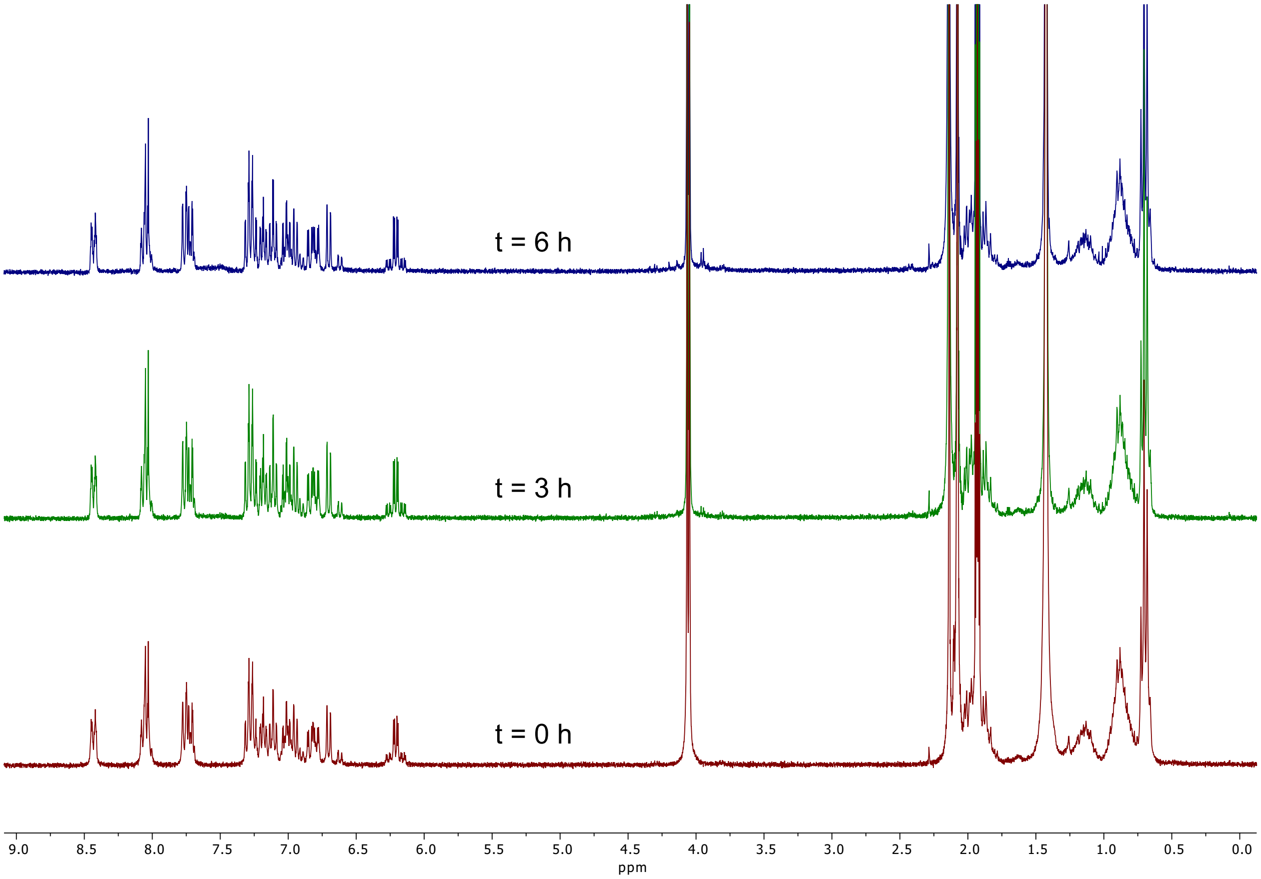

Figure S15. ^1H NMR spectra of complex **4a** (CD_3CN, 300 MHz) after different times of irradiation.

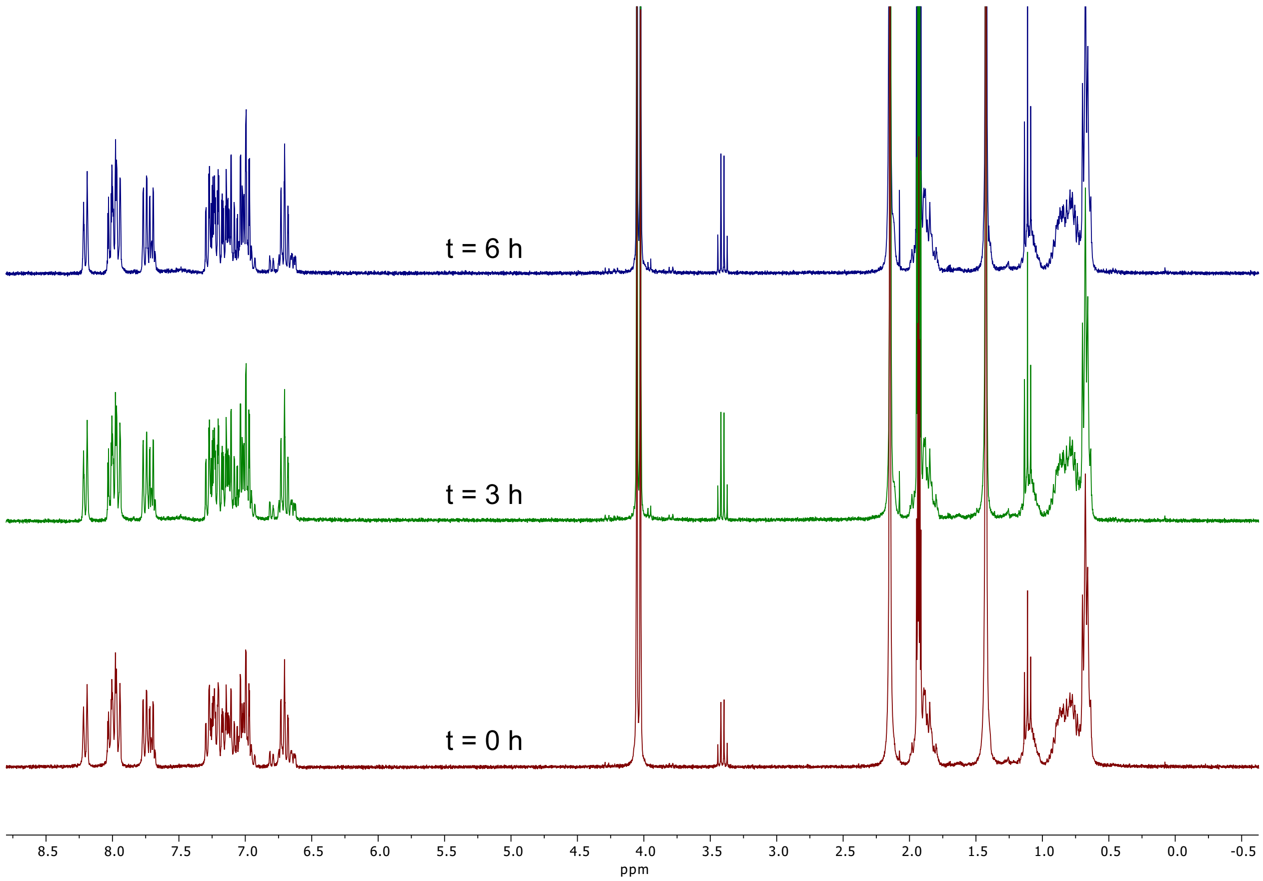

Figure S16. ^{1}H NMR spectra of complex **4b** (CD_3CN, 300 MHz) after different times of irradiation.

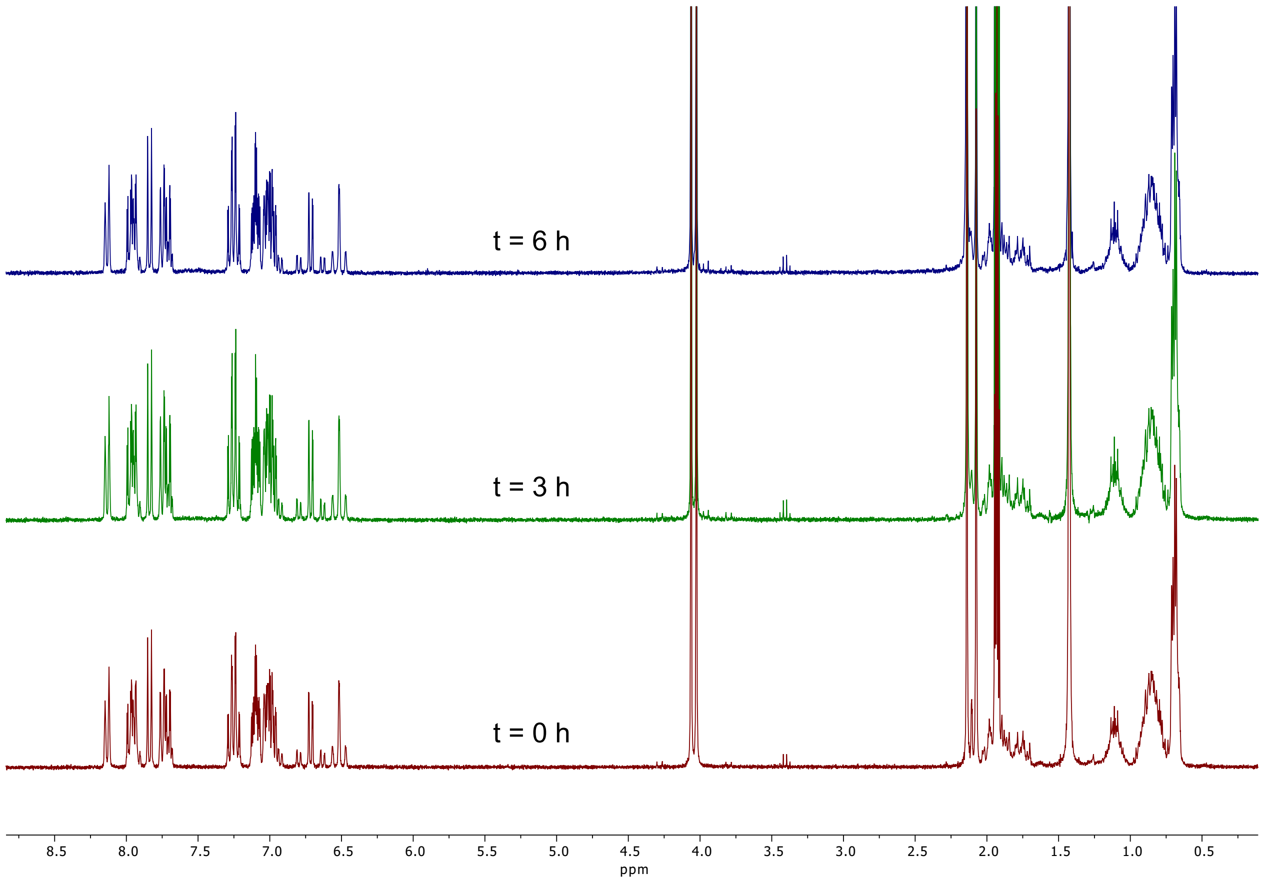

Figure S17. ^{1}H NMR spectra of complex **4c** (CD_3CN, 300 MHz) after different times of irradiation.

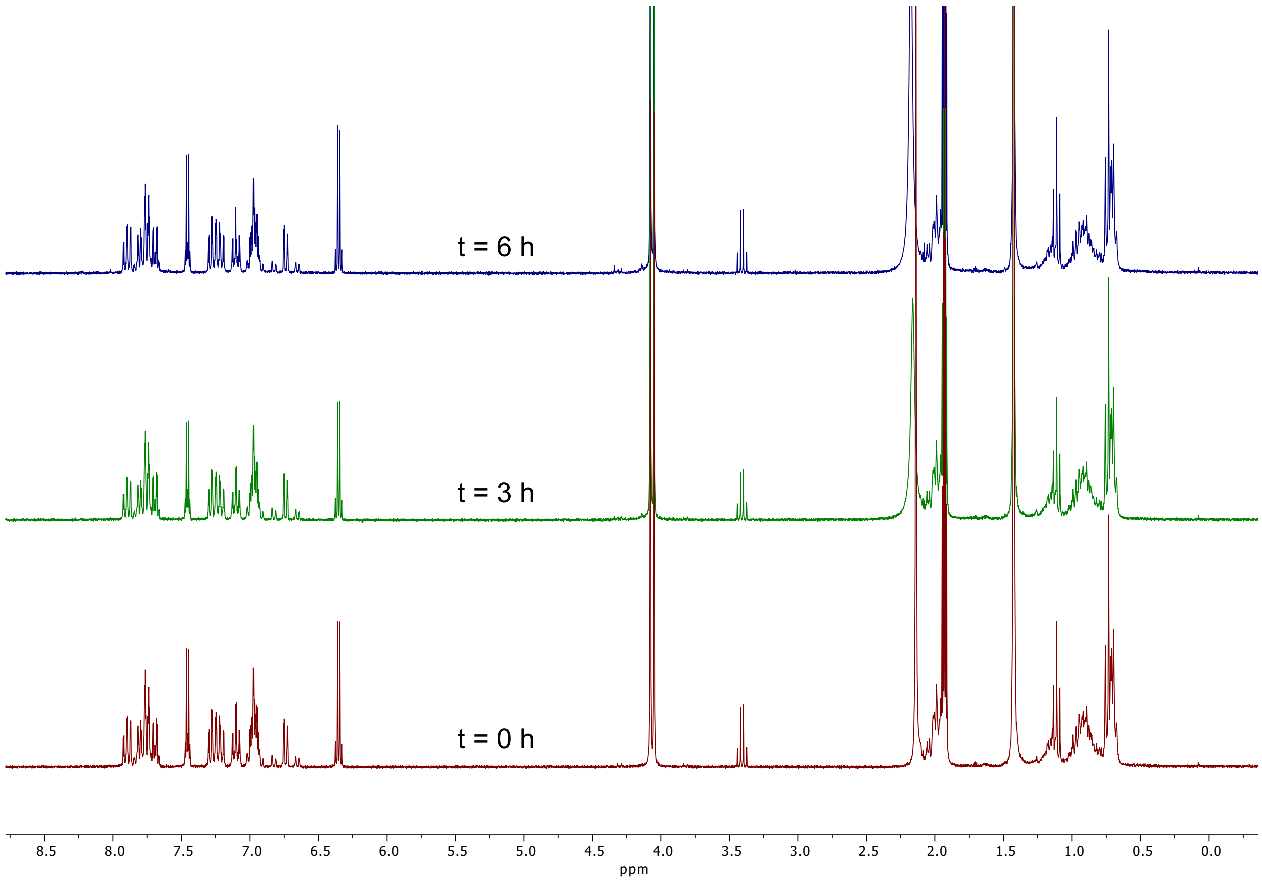

Figure S18. ^{1}H NMR spectra of complex **4d** (CD_3CN, 300 MHz) after different times of irradiation.

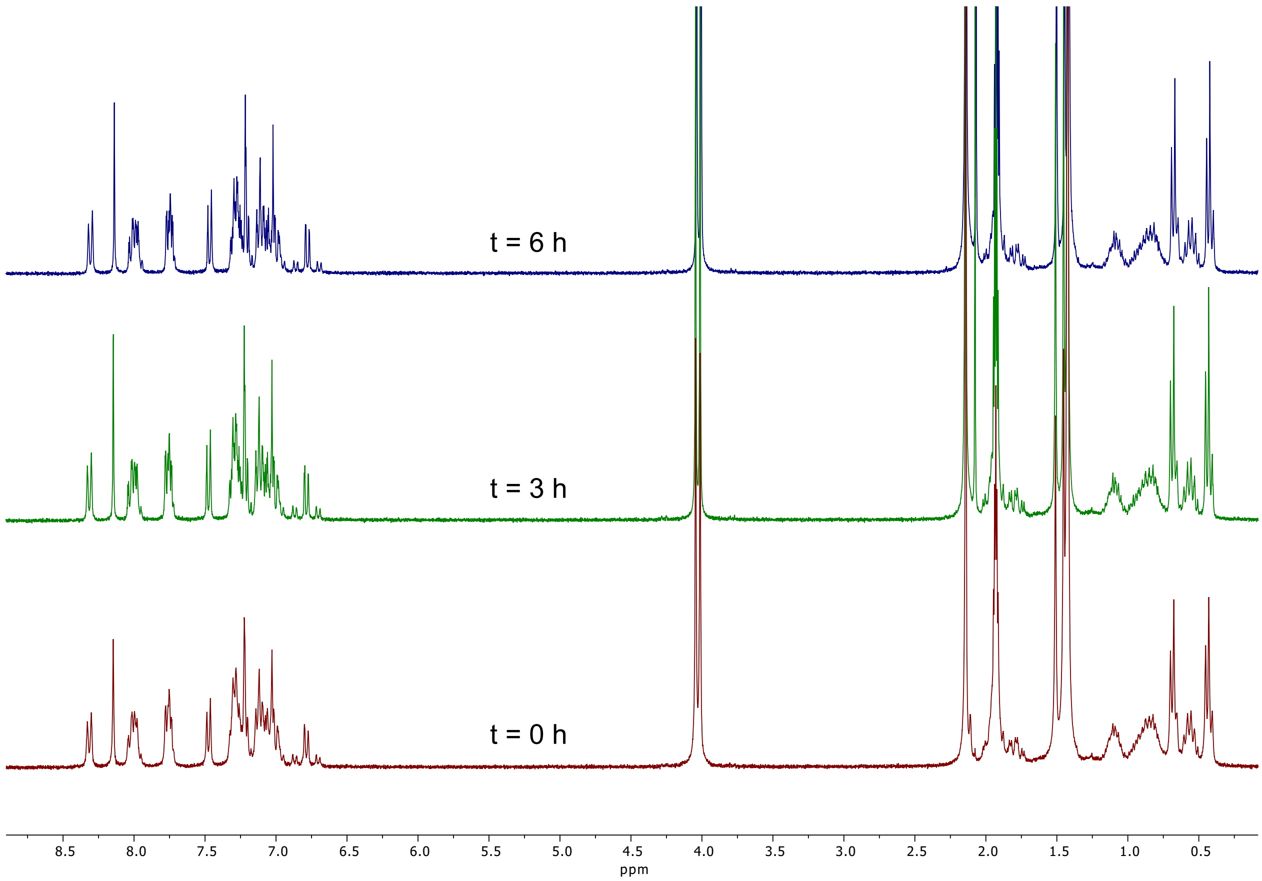

Figure S19. ^{1}H NMR spectra of complex **4e** (CD_3CN, 300 MHz) after different times of irradiation.

5. Excitation and emission spectra

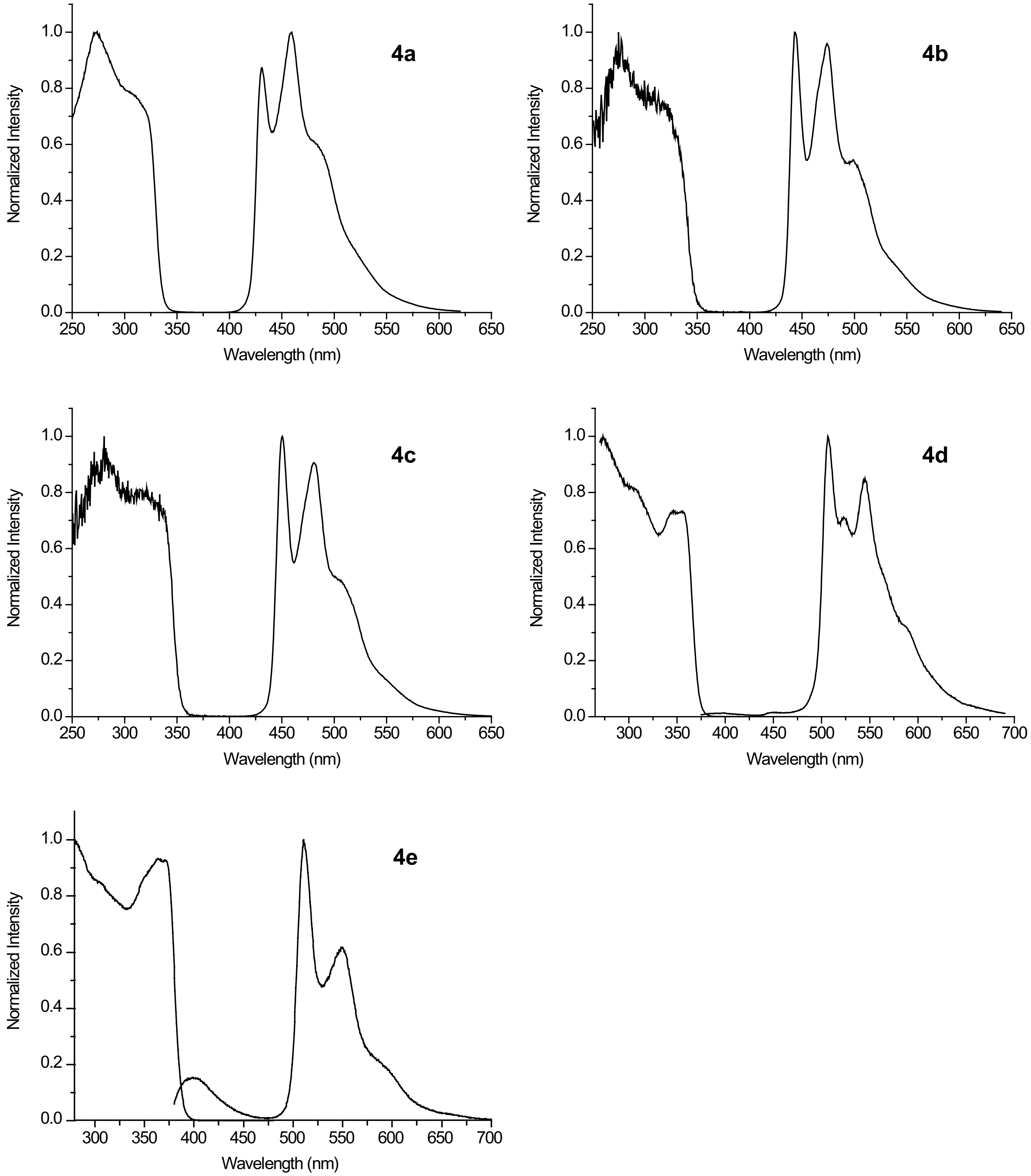

Figure S20. Excitation and emission spectra of complexes **4a-e** in CH_2Cl_2 at 298 K. The collected λ_{em} of the excitation spectra corresponds in all cases to the highest-energy peak of the phosphorescence emission.

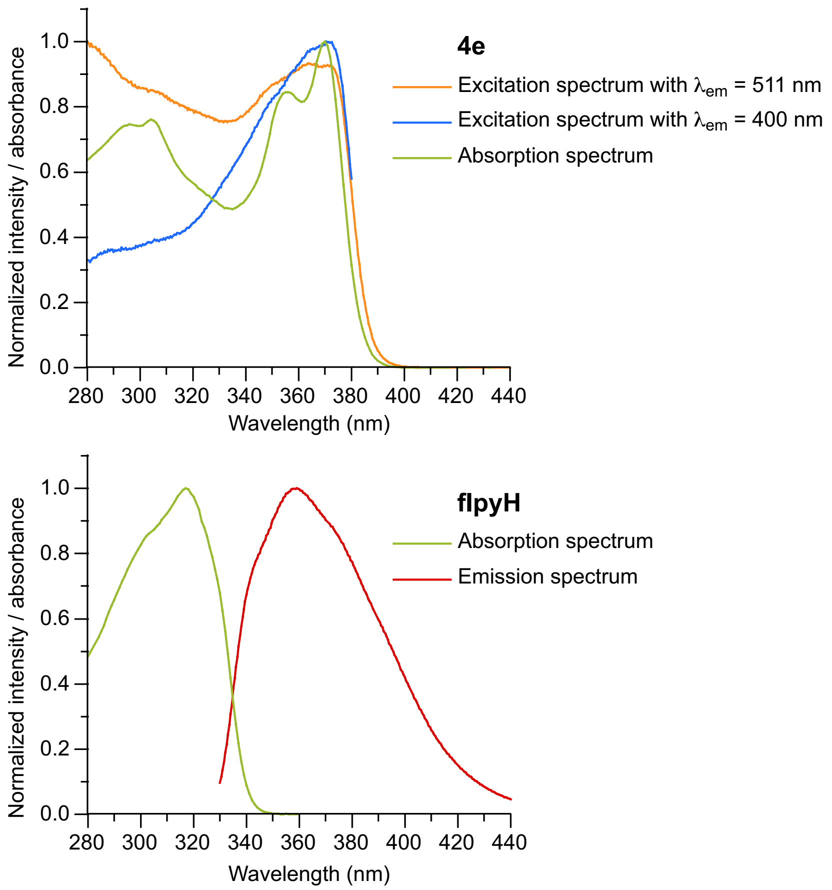

Figure S21. Top: Excitation spectra of complex **4e** monitored at different emission wavelengths compared with the absorption spectrum in CH_2Cl_2 at 298 K. **Bottom:** Absorption and emission spectra of 2-(9,9-dimethylfluoren-2-yl)pyridine (flpyH). **Comment:** The excitation spectrum of **4e** monitored at λ_{em} = 400 nm and the fluorescence emission are significantly red-shifted with respect to the absorption and emission spectra of flpyH, and therefore the observed fluorescence band does not arise from free flpyH. The excitation spectrum monitored at λ_{em} = 400 nm coincides with the one at λ_{em} = 511 nm in the lowest-energy features, but it is different in the region at higher energies. This can be a consequence of relatively low internal conversion rates between higher-lying ^{1}LC(trz) states and the lowest ^{1}LC(flpy) state, possibly in combination with different intersystem crossing rates to the triplet manifold depending on the nature of the singlet state. A related behavior has been previously observed by us in heteroleptic tris-cyclometalated Pt(IV) complexes of the type *fac*-$[Pt(C^\wedge N)_2(C'^\wedge N')]^+$ [3] and by other authors in Os(II) complexes.[4]

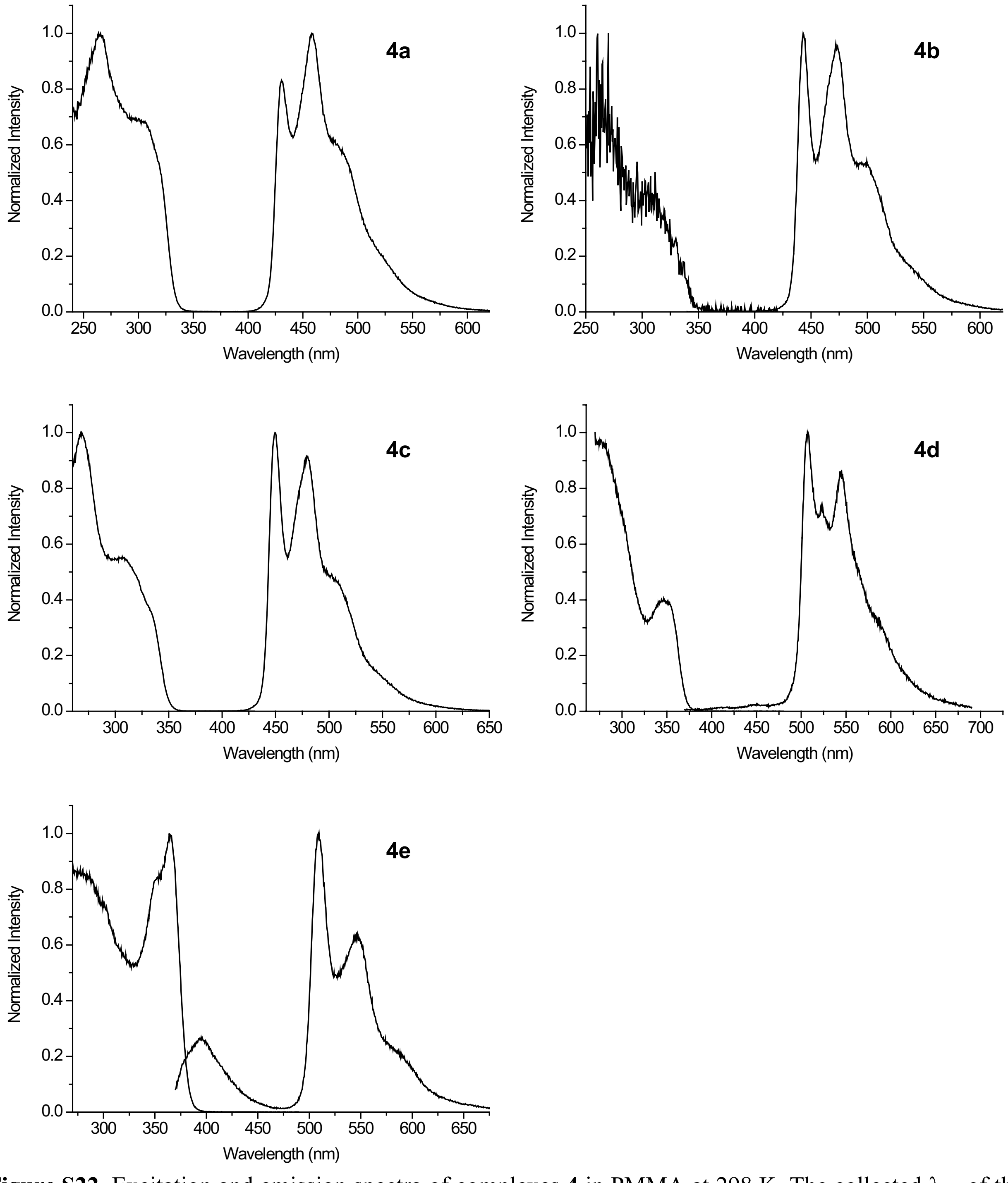

Figure S22. Excitation and emission spectra of complexes **4** in PMMA at 298 K. The collected λ_{em} of the excitation spectra corresponds in all cases to the highest-energy peak of the phosphorescence emission.

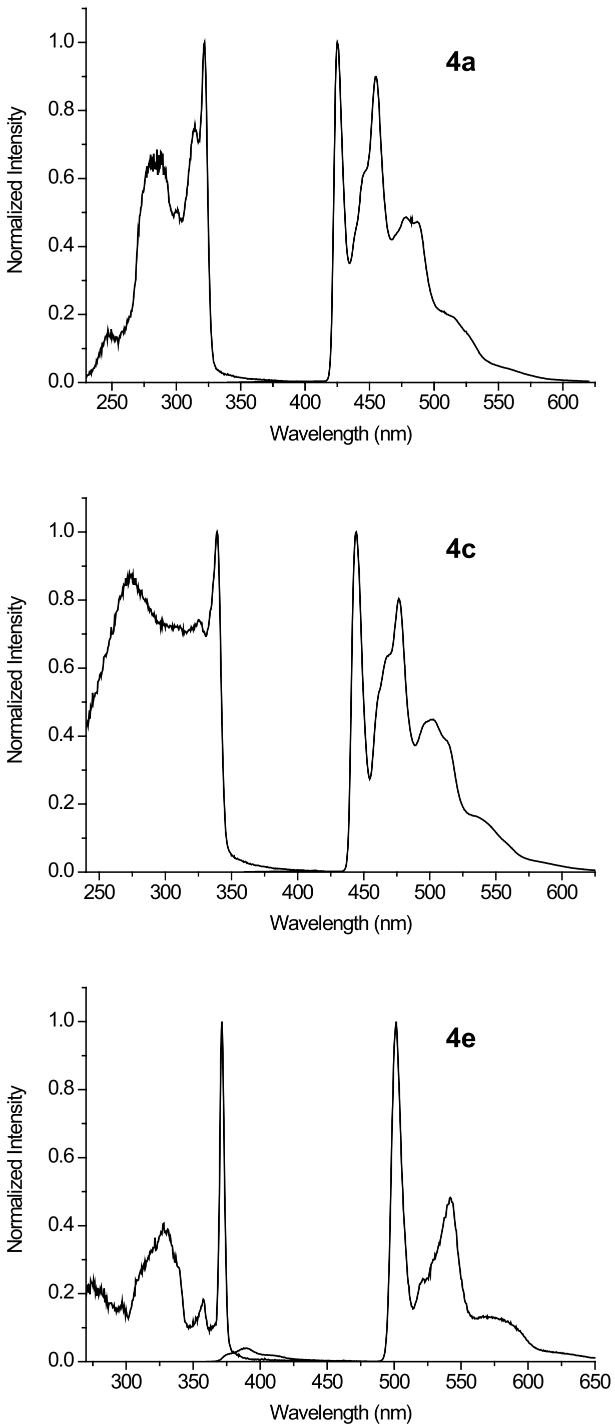

Figure S23. Excitation and emission spectra of complexes **4** in PrCN at 77 K. The collected λ_{em} of the excitation spectra corresponds in all cases to the highest-energy peak of the phosphorescence emission.

6. Electrochemical measurements

Cyclic voltammograms (Figures 5 and S24) were registered at 298 K using a potentiostat/galvanostat AUTOLAB-100 (Echo-Chemie, Utrecht) and a three-electrode electrochemical cell equipped with a glassy carbon working electrode (Metrohm, 2 mm diameter), an Ag/AgCl/3 M KCl electrode reference, and a glassy carbon rod counter electrode. The measurements were carried out under an argon atmosphere, using degassed 1 mM solutions of the complexes in extra-dry MeCN (Acros Organics) and 0.1 M $(Bu_4N)PF_6$ as the electrolyte. The working electrode was polished with alumina slurry (0.05 μm) and rinsed with water and acetone before each experiment. The electrodes were activated electrochemically in the background solution by means of several voltammetric cycles at 1 V s^{-1} between –2.8 V and 2.2 V. The reference electrode was checked against the Fc^{+}/Fc redox couple. Potentials are given against the standard calomel electrode (SCE).

The HOMO/LUMO energies were estimated from the onset values of the oxidation and reduction waves, respectively, referenced against Fc^{+}/Fc (0.40 V vs SCE in MeCN), using a formal potential of 5.1 eV for the Fc^{+}/Fc couple in the Fermi scale:[5]

$$E_{\mathrm{HOMO}} = -(E_{onset,ox} + 5.1 - 0.4)\ \mathrm{eV};\ E_{\mathrm{LUMO}} = -(E_{onset,red} + 5.1 - 0.4)\ \mathrm{eV}$$

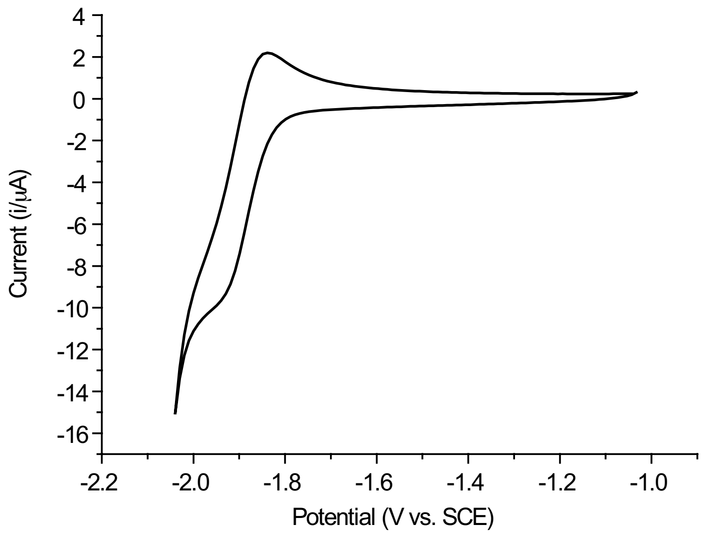

Figure S24. Cyclic voltammogram of complex **4e** showing the first reduction wave.

7. Computational methods and data

The DFT calculations on complex **4c** were carried out with Gaussian 09,[6] using the B3LYP functional[7,8] together with the 6-31G**[9,10] basis set for the light atoms and the LANL2DZ[11] basis set and effective core potential for the Pt atom. Geometry optimizations were carried out without symmetry restrictions, using "tight" convergence criteria and "ultrafine" integration grid. The geometry of the lowest triplet excited state was optimized through spin-unrestricted DFT (UB3LYP), by setting a triplet multiplicity. Vertical excitation energies were obtained from TDDFT calculations at the ground-state geometry. The solvent effect (CH_2Cl_2) was accounted for in all cases by using the integral equation formalism variant of the polarizable continuum solvation model (IEFPCM).[12] The optimized geometries were confirmed as minima on the potential energy surface by frequency calculations (zero imaginary frequencies). Natural spin densities were calculated using the NBO 5.9 program.[13]

Table S2. Fragment contributions (%; from atomic orbital contributions) to the frontier orbitals of **4c** in CH_2Cl_2 solution.

energy (a.u.)	number	L1	L2	L3	Pt
–0.016	174 (LUMO+5)	8	87	0	4
–0.031	173 (LUMO+4)	31	23	14	32
–0.050	172 (LUMO+3)	1	0	97	1
–0.069	171 (LUMO+2)	48	38	12	1
–0.070	170 (LUMO+1)	10	3	85	1
–0.072	169 (LUMO)	39	57	2	2
–0.235	168 (HOMO)	1	0	95	3
–0.240	167 (HOMO–1)	25	41	25	8
–0.248	166 (HOMO–2)	85	3	7	6
–0.250	165 (HOMO–3)	8	7	80	5
–0.255	164 (HOMO–4)	11	69	16	4
–0.260	163 (HOMO–5)	93	3	1	3

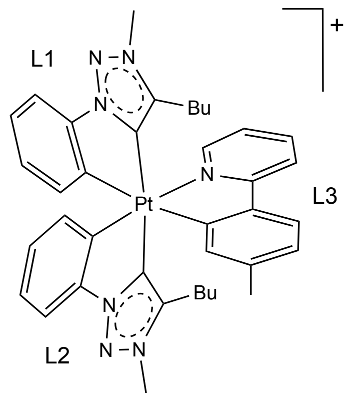

Figure S25. Ligand numbering in complex **4c**.

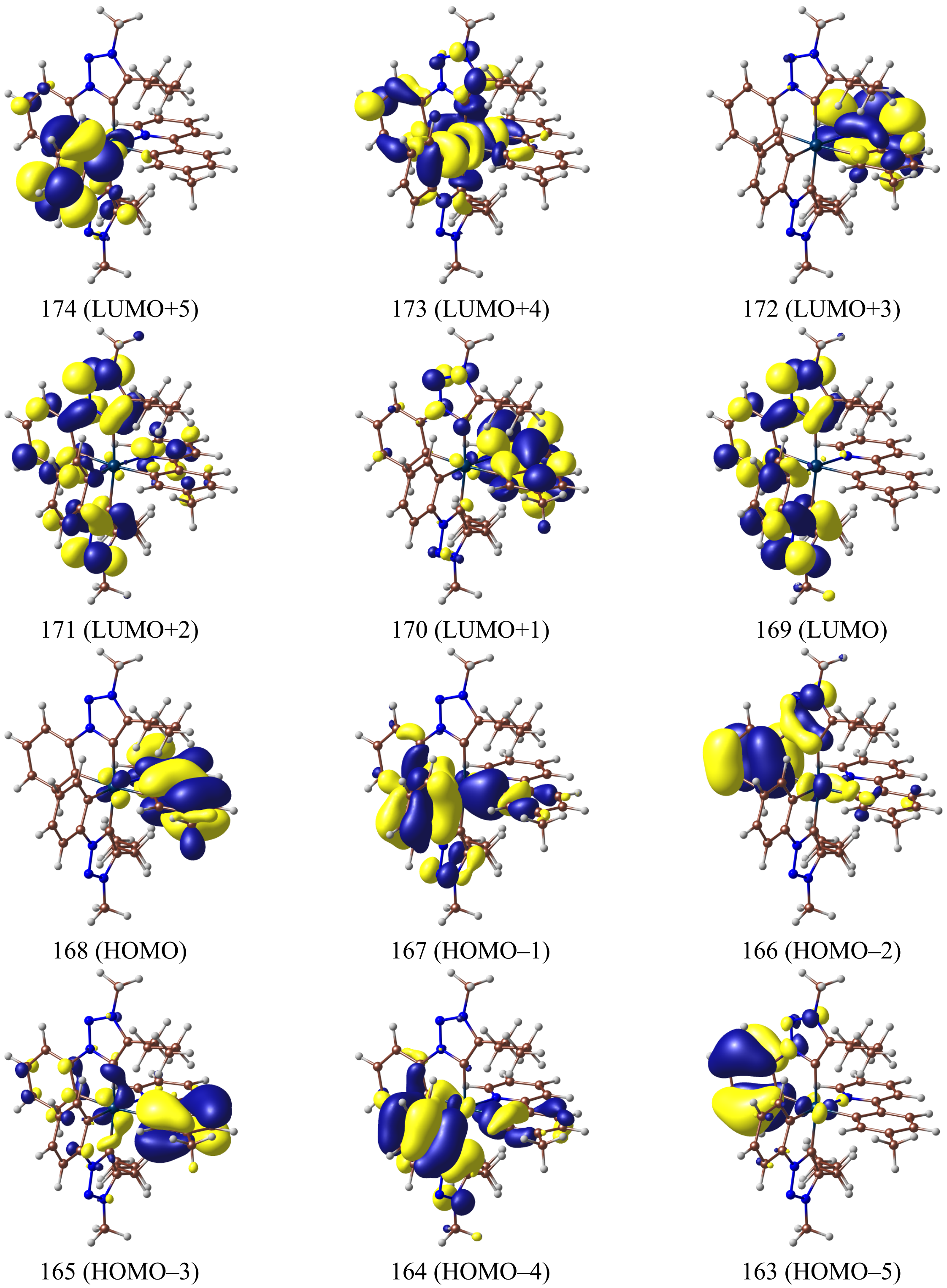

Figure S26. Molecular orbital isosurfaces of **4c** (0.03 e $bohr^{-3}$).

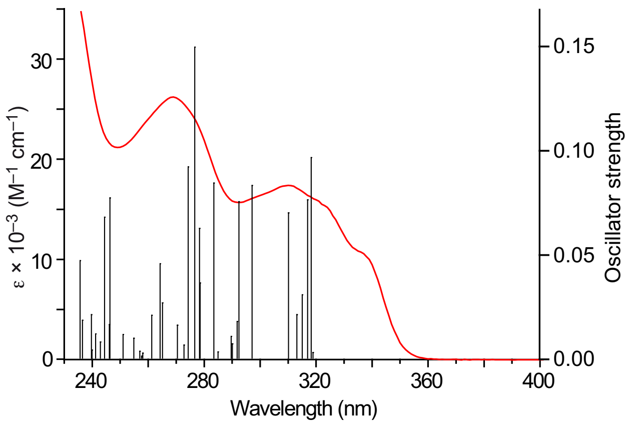

Figure S27. Calculated stick absorption spectrum of **4c** compared with the experimental spectrum in CH_2Cl_2 solution (*ca.* 1×10^{-5} M) at 298 K.

Table S3. Selected vertical singlet excitations of **4c** from TDDFT calculations at the ground state geometry in CH_2Cl_2 solution.

State	Monoexcitations	ΔE (eV)	λ (nm)	Oscillator strength	Main character
S1	167 ->169 (8%)<br>167 ->171 (3%)<br>168 ->169 (86%)	3.894	318.4	0.0027	LLCT
S2	167 ->169 (37%)<br>167 ->171 (7%)<br>168 ->169 (4%)<br>168 ->170 (41%)<br>168 ->171 (6%)	3.902	317.7	0.0962	LC(tpy)
S3	167 ->169 (19%)<br>167 ->170 (31%)<br>167 ->171 (4%)<br>168 ->169 (5%)<br>168 ->170 (35%)	3.918	316.5	0.076	LC(tpy)
S4	164 ->170 (2%)<br>167 ->169 (5%)<br>167 ->170 (58%)<br>167 ->171 (15%)<br>168 ->170 (14%)	3.941	314.6	0.0303	LLCT
S6	167 ->169 (24%)<br>167 ->170 (4%)<br>167 ->171 (64%)	4.005	309.6	0.0697	LC(trz)
S7	163 ->169 (2%)<br>165 ->169 (2%)<br>166 ->169 (57%)<br>166 ->170 (8%)<br>166 ->171 (19%)	4.178	296.8	0.0828	LC(trz)
S8	165 ->170 (47%)<br>165 ->171 (8%)<br>166 ->170 (21%)<br>166 ->171 (4%)<br>168 ->172 (13%)	4.247	291.9	0.0749	LC(tpy)
S9	165 ->169 (19%)<br>166 ->169 (31%)<br>166 ->170 (9%)<br>166 ->171 (34%)	4.255	291.4	0.0175	LC(trz)

S10	165 ->169 (57%)<br>165 ->170 (7%)<br>165 ->171 (3%)<br>166 ->169 (4%)<br>166 ->170 (24%)	4.282	289.6	0.007	LLCT
S11	164 ->170 (2%)<br>165 ->169 (11%)<br>165 ->170 (11%)<br>165 ->171 (9%)<br>166 ->170 (30%)<br>166 ->171 (30%)	4.287	289.2	0.0106	LLCT
S13	162 ->169 (3%)<br>164 ->169 (78%)<br>164 ->171 (5%)<br>165 ->171 (5%)	4.382	283.0	0.0841	LC(trz)
S15	164 ->169 (2%)<br>164 ->170 (26%)<br>164 ->171 (39%)<br>165 ->170 (8%)<br>168 ->172 (15%)	4.462	277.9	0.0622	LC(trz)
S16	164 ->170 (11%)<br>164 ->171 (9%)<br>165 ->170 (4%)<br>168 ->170 (2%)<br>168 ->172 (62%)	4.490	276.2	0.1491	LC(tpy)
S17	163 ->169 (67%)<br>163 ->170 (6%)<br>163 ->171 (15%)<br>166 ->169 (3%)<br>166 ->173 (2%)	4.529	273.8	0.0917	LC(trz)
S19	163 ->169 (26%)<br>163 ->170 (11%)<br>163 ->171 (59%)	4.594	269.9	0.0158	LC(trz)
S21	167 ->173 (86%)<br>167 ->175 (2%)<br>168 ->173 (3%)	4.683	264.7	0.0265	LMCT

Table S4. Lowest-energy vertical triplet excitations of **4c** from TDDFT calculations at the ground state geometry in CH_2Cl_2 solution.

State	Monoexcitations	ΔE (eV)	λ (nm)	Main character
T1	165 ->170 (2%)<br>168 ->170 (61%)<br>168 ->171 (13%)<br>168 ->172 (8%)	2.920	424.6	LC(tpy)
T2	160 ->169 (3%)<br>162 ->169 (9%)<br>162 ->171 (4%)<br>163 ->171 (3%)<br>164 ->169 (15%)<br>164 ->171 (4%)<br>165 ->169 (2%)<br>166 ->169 (4%)<br>166 ->171 (8%)<br>167 ->169 (14%)<br>167 ->171 (12%)<br>167 ->174 (3%)	3.197	387.8	LC(trz)
T3	162 ->171 (2%)<br>163 ->169 (8%)<br>163 ->171 (4%)<br>164 ->171 (6%)<br>165 ->171 (2%)<br>166 ->169 (19%)<br>166 ->170 (5%)<br>166 ->171 (17%)<br>167 ->169 (8%)	3.200	387.5	LC(trz)
T15	163 ->169 (4%)<br>163 ->170 (2%)<br>163 ->171 (8%)<br>163 ->173 (5%)<br>163 ->175 (7%)<br>164 ->169 (3%)<br>166 ->169 (5%)<br>166 ->173 (19%)<br>166 ->174 (3%)<br>166 ->175 (14%)<br>167 ->173 (2%)<br>167 ->174 (4%)	4.181	296.5	LC(trz)/LMCT

Table S5. Energies, free energies, enthalpies and entropies of the optimized structures of **4c** in the ground state (S_0) and the first triplet excited state (T_1) in CH_2Cl_2 solution.[a]

	E_0 [b]	ZPE [c]	G [d]	H [e]	S [f]
**S_0**	-1975.639759	-1974.905265	-1974.984948	-1974.860579	261.756
**T_1**	-1975.536681	-1974.806778	-1974.887321	-1974.761476	264.862

[a] Thermal corrections from vibrational calculations at 298.15 K. [b] Electronic energy (Hartrees). [c] Sum of electronic and zero-point energies (Hartrees). [d] Free Energy (Hartrees). [e] Enthalpy (Hartrees). [f] Entropy (cal mol^{-1} K^{-1}).

Table S6. Cartesian coordinates (Å) of the optimized structure of **4c** in the ground state (S_0) and the first triplet excited state (T_1) at the B3LYP(6-31G**+LANL2DZ) level in CH_2Cl_2 solution.

S_0
C -49.038045653 21.001152887 -81.334524121
C -48.576028776 20.068420728 -82.254774470
C -47.613927968 20.188068181 -83.395031847
C -48.287568913 20.538930001 -84.739350555
C -47.275438947 20.638226936 -85.886805802
C -47.926833386 20.996106864 -87.226144952
C -49.088358344 17.590153599 -82.598324807
C -50.623460480 20.884342883 -79.486980074
C -50.300733373 22.238749932 -79.306287740
C -50.954968436 22.885958367 -78.251648595
C -51.868342073 22.206950507 -77.434358677
C -52.154516314 20.856471964 -77.654945684
C -51.526236708 20.174429642 -78.697153396
C -48.927650653 24.927151759 -80.053173801
C -48.364117789 25.859894236 -79.190663040
C -47.267462711 25.739094553 -78.179153958
C -47.765519787 25.386381354 -76.760668413
C -46.615029551 25.238827051 -75.757879642
C -47.097707995 24.896355231 -74.345037243
C -48.858073476 28.329984677 -78.770331730
C -50.710181941 25.016211232 -81.676608108
C -50.388581453 23.661070108 -81.868302566
C -51.138626703 22.969360247 -82.823529943
C -52.151264996 23.614724182 -83.543393568
C -52.440188471 24.964668203 -83.324097754
C -51.712565362 25.683492259 -82.377210210
C -47.311248689 23.486152050 -82.033641352
C -47.428125316 24.063061034 -83.300492744
C -46.309299723 24.366196622 -84.092903924
C -45.033213129 24.067551526 -83.589197034
C -44.884447117 23.497273927 -82.330711405
C -46.012254330 23.205061932 -81.541478993
C -45.904643279 22.601569041 -80.205320198
C -44.700845419 22.241619713 -79.576207825
C -44.718564178 21.680000724 -78.306300434
C -45.941692565 21.474781236 -77.661669939
C -47.102883348 21.845266377 -78.325814374
H -50.756795207 23.936263074 -78.055914927
H -52.360550629 22.735285570 -76.622364234
H -52.864186556 20.334846868 -77.020626736
H -51.730871561 19.126802520 -78.890064962
H -50.939179480 21.921638719 -83.017668672
H -52.718810665 23.055349433 -84.281492112
H -53.227566117 25.455953557 -83.886324768
H -51.912825136 26.731854303 -82.184868038
H -48.415222499 24.284868165 -83.698208077
H -44.152734896 24.286834058 -84.187254640
H -43.882757660 23.282563380 -81.970857117
H -43.757710747 22.400828933 -80.083733787
H -43.787553751 21.402440060 -77.822413349
H -45.999453955 21.037025918 -76.672074087
H -48.082632852 21.712404152 -77.882079672
H -46.578020164 24.964497967 -78.524559591
H -46.691847143 26.671293989 -78.145397212
H -48.340303384 24.453081653 -76.806958722
H -48.461135240 26.159685689 -76.409121678
H -45.928641943 24.458381151 -76.112278642
H -46.034632548 26.170746288 -75.733079751
H -47.759824888 25.676282749 -73.952481918
H -47.654514646 23.952510456 -74.334465869
H -46.256022475 24.794641066 -73.652666493
H -48.984024382 28.238821672 -77.690766221
H -47.857776549 28.702482015 -78.997573141
H -49.606162728 29.008400388 -79.175163439
H -46.891319395 20.967273901 -83.141824238
H -47.049804965 19.253847182 -83.498687616
H -48.817047264 21.492873330 -84.629437266
H -49.048647455 19.786011954 -84.984442173
H -46.519120249 21.391801480 -85.631291509
H -46.739891145 19.683980283 -85.980966014
H -48.438276212 21.963784830 -87.171406858
H -47.181466487 21.057983348 -88.025450253
H -48.668844243 20.244929387 -87.519770062
H -49.813198087 16.920297913 -82.140272684
H -49.288615473 17.680658308 -83.666473846
H -48.078096004 17.207323282 -82.441570863
N -49.924279377 20.275726921 -80.573869397
N -50.053713910 19.009558591 -80.927375675
N -49.228128661 18.896610734 -81.956475138
N -49.902954434 25.646928863 -80.694854133
N -50.001123216 26.912944632 -80.325071422
N -49.057015705 27.026992119 -79.403745256
N -47.082089116 22.392632136 -79.553227774
Pt -48.855658735 22.968868324 -80.705663637
C -46.470079012 25.023929123 -85.442371527

H	-46.391060572	26.115205061	-85.357360789
H	-45.694154191	24.698741078	-86.141933796
H	-47.446498456	24.802237141	-85.88252211

T_1

$\langle S^2 \rangle = 2.02745$

C	-49.048379374	21.000832840	-81.337693893
C	-48.581262815	20.067018144	-82.253855818
C	-47.620847067	20.188966353	-83.394985809
C	-48.297151413	20.530073584	-84.740237986
C	-47.287103231	20.631180148	-85.889425229
C	-47.943287782	20.973987573	-87.230369904
C	-49.075426205	17.583305728	-82.584371461
C	-50.629033774	20.882816101	-79.487681763
C	-50.310364928	22.238837881	-79.311769487
C	-50.965701103	22.887633107	-78.258877686
C	-51.876523206	22.208621331	-77.438832870
C	-52.158838210	20.856542133	-77.654529221
C	-51.529167344	20.172949851	-78.694805966
C	-48.944477114	24.927325858	-80.054098803
C	-48.378434891	25.858032485	-79.191534595
C	-47.276898153	25.733245134	-78.185968174
C	-47.767852718	25.372582210	-76.767120671
C	-46.612424993	25.218114419	-75.771004297
C	-47.088419981	24.868933848	-74.357540114
C	-48.871898708	28.326815434	-78.763855659
C	-50.728108085	25.020277694	-81.677354248
C	-50.404698340	23.666398870	-81.875510489
C	-51.154753538	22.979198605	-82.834135714
C	-52.168754251	23.626657288	-83.550310444
C	-52.459208424	24.975118343	-83.323827873
C	-51.731794287	25.690092528	-82.373731399
C	-47.340811811	23.495099749	-82.042891899
C	-47.435901311	24.067300008	-83.287999398
C	-46.298733906	24.382711874	-84.085425062
C	-44.995765266	24.081995789	-83.555807008
C	-44.831721378	23.519267856	-82.325398994
C	-45.990733538	23.202821819	-81.499692511
C	-45.894036079	22.635222068	-80.229160691
C	-44.665953800	22.272989182	-79.561875589
C	-44.683491792	21.715092266	-78.310358165
C	-45.932164200	21.493557079	-77.668437587
C	-47.109844199	21.855448964	-78.343276165
H	-50.770083589	23.938946161	-78.066377820
H	-52.369610408	22.738260287	-76.628226460
H	-52.866490964	20.334962599	-77.017923170
H	-51.730619090	19.124052333	-78.884118598
H	-50.954121223	21.932412133	-83.033139698
H	-52.736274096	23.070516765	-84.290919405
H	-53.247672207	25.468310798	-83.882910516
H	-51.933506441	26.737205132	-82.176050887
H	-48.416579469	24.291685449	-83.700259697
H	-44.122213593	24.315147316	-84.159646934
H	-43.832104125	23.310713281	-81.961223974
H	-43.723022385	22.445991988	-80.066318564
H	-43.757379059	21.443865960	-77.814953008
H	-45.996252645	21.051982002	-76.681517784
H	-48.080138469	21.699784544	-77.882458033
H	-46.589496166	24.960701755	-78.540312668
H	-46.702234522	26.666007244	-78.149939670
H	-48.343385524	24.439903763	-76.816056337
H	-48.461256263	26.144297532	-76.407593414
H	-45.929472857	24.438193462	-76.132966827
H	-46.030351874	26.148974507	-75.744269204
H	-47.747816903	25.647425602	-73.957547732
H	-47.646033469	23.925541837	-74.349185993
H	-46.243399693	24.762685269	-73.669910263
H	-48.996029562	28.232641146	-77.684319784
H	-47.871888171	28.699852374	-78.991539462
H	-49.620555767	29.006478046	-79.165557284
H	-46.903148465	20.974170827	-83.145623730
H	-47.048947433	19.259169329	-83.495232088
H	-48.833618185	21.480526619	-84.633715135
H	-49.053113817	19.770752070	-84.981232713
H	-46.536917115	21.393237032	-85.640269001
H	-46.742727323	19.681437798	-85.977839106
H	-48.465137567	21.936316403	-87.181241088
H	-47.199328865	21.038566935	-88.030766897
H	-48.677374316	20.212983856	-87.518394201
H	-49.791659780	16.909265520	-82.118993938
H	-49.281378180	17.665327302	-83.652163306
H	-48.060821330	17.211162939	-82.429898801
N	-49.928780347	20.273241164	-80.572946512
N	-50.050102751	19.004334394	-80.920153464
N	-49.224643288	18.892052507	-81.949446605
N	-49.920840360	25.648104060	-80.692820505
N	-50.018134677	26.913435126	-80.320050059
N	-49.071868970	27.025571648	-79.400408793
N	-47.118356650	22.400416651	-79.550086578
Pt	-48.870320225	22.970123268	-80.711661332
C	-46.431223114	25.023100537	-85.433767105
H	-45.995077025	26.032188263	-85.439394905
H	-45.892946292	24.453266918	-86.202365308
H	-47.477065269	25.108280876	-85.739174875

8. References

(1) Sheldrick, G. M. A Short History of SHELX. *Acta Crystallogr., Sect. A Found. Crystallogr.* **2008**, *64*, 112–122.

(2) Sheldrick, G. M. SHELXT – Integrated Space-Group and Crystal-Structure Determination. *Acta Crystallogr. Sect. A Found. Adv.* **2015**, *71*, 3–8.

(3) López-López, J. C.; Bautista, D.; González-Herrero, P. Stereoselective Formation of Facial Tris-Cyclometalated Pt(IV) Complexes: Dual Phosphorescence from Heteroleptic Derivatives. *Chem. Eur. J.* **2020**, *26*, 11307–11315.

(4) Hsu, C. C.; Lin, C. C.; Chou, P. T.; Lai, C. H.; Hsu, C. W.; Lin, C. H.; Chi, Y. Harvesting Highly Electronically Excited Energy to Triplet Manifolds: State-Dependent Intersystem Crossing Rate in Os(II) and Ag(I) Complexes. *J. Am. Chem. Soc.* **2012**, *134*, 7715–7724.

(5) Cardona, C. M.; Li, W.; Kaifer, A. E.; Stockdale, D.; Bazan, G. C. Electrochemical Considerations for Determining Absolute Frontier Orbital Energy Levels of Conjugated Polymers for Solar Cell Applications. *Adv. Mater.* **2011**, *23*, 2367–2371.

(6) Frisch, M. J.; Trucks, G. W.; Schlegel, H. B.; Scuseria, G. E.; Robb, M. A.; Cheeseman, J. R.; Scalmani, G.; Barone, V.; Mennucci, B.; Petersson, G. A.; et al. Gaussian 09 (Revision A.02). Gaussian Inc.: Wallingford CT 2009.

(7) Lee, C. T.; Yang, W. T.; Parr, R. G. Development of The Colle-Salvetti Correlation-Energy Formula into a Functional of the Electron-Density. *Phys. Rev. B* **1988**, *37*, 785–789.

(8) Becke, A. Density Functional Thermochemistry III The Role of Exact Exchange. *J. Chem. Phys.* **1993**, *98*, 5648–5652.

(9) Hariharan, P. C.; Pople, J. A. Influence of Polarization Functions on Molecular-Orbital Hydrogenation Energies. *Theor. Chim. Acta* **1973**, *28*, 213–222.

(10) Francl, M. M.; Pietro, W. J.; Hehre, W. J.; Binkley, J. S.; Gordon, M. S.; Defrees, D. J.; Pople, J. A. Self-Consistent Molecular-Orbital Methods. 23. A Polarization-Type Basis Set for 2nd-Row Elements. *J. Chem. Phys.* **1982**, *77*, 3654–3665.

(11) Hay, P. J.; Wadt, W. R. Ab Initio Effective Core Potentials for Molecular Calculations–Potentials for K to Au Including the Outermost Core Orbitals. *J. Chem. Phys.* **1985**, *82*, 299–310.

(12) Tomasi, J.; Mennucci, B.; Cammi, R. Quantum Mechanical Continuum Solvation Models. *Chem. Rev.* **2005**, *105*, 2999–3093.

(13) Glendening, E. D.; Badenhoop, J. K.; A. E. Reed; Carpenter, J. E.; Bohmann, J. A.; Morales, C. M.; Weinhold, F. NBO 5.9. Theoretical Chemistry Institute, University of Wisconsin: Madison, WI 2009.
